# Are we overestimating the niche? Removing marginal localities helps ecological niche models detect environmental barriers

**DOI:** 10.1002/ece3.1900

**Published:** 2016-01-28

**Authors:** Mariano Soley‐Guardia, Eliécer E. Gutiérrez, Darla M. Thomas, José Ochoa‐G, Marisol Aguilera, Robert P. Anderson

**Affiliations:** ^1^ Department of BiologyCity College of New YorkCity University of New YorkNew YorkNew York; ^2^ The Graduate CenterCity University of New YorkNew YorkNew York; ^3^ Department of Vertebrate ZoologyDivision of MammalsNational Museum of Natural HistorySmithsonian InstitutionWashingtonDistrict of Columbia; ^4^ Cabañas BougainvillaeLos TaquesVenezuela; ^5^ Departamento de Estudios AmbientalesUniversidad Simón BolívarCaracasVenezuela; ^6^ Division of Vertebrate Zoology (Mammalogy)American Museum of Natural HistoryNew YorkNew York

**Keywords:** Gallery forests, habitat connectivity, niche conservatism, Paraguaná, small mammals, soft allopatry

## Abstract

Correlative ecological niche models (ENMs) estimate species niches using occurrence records and environmental data. These tools are valuable to the field of biogeography, where they are commonly used to infer potential connectivity among populations. However, a recent study showed that when locally relevant environmental data are not available, records from patches of suitable habitat protruding into otherwise unsuitable regions (e.g., gallery forests within dry areas) can lead to overestimations of species niches and their potential distributions. Here, we test whether this issue obfuscates detection of an obvious environmental barrier existing in northern Venezuela – that of the hot and xeric lowlands separating the Península de Paraguaná from mainland South America. These conditions most likely promote isolation between mainland and peninsular populations of three rodent lineages occurring in mesic habitat in this region. For each lineage, we calibrated optimally parameterized ENMs using mainland records only, and leveraged existing habitat descriptions to assess whether those assigned low suitability values corresponded to instances where the species was collected within locally mesic conditions amidst otherwise hot dry areas. When this was the case, we built an additional model excluding these records. We projected both models onto the peninsula and assessed whether they differed in their ability to detect the environmental barrier. For the two lineages in which we detected such problematic records, only the models built excluding them detected the barrier, while providing additional insights regarding peninsular populations. Overall, the study reveals how a simple procedure like the one applied here can deal with records problematic for ENMs, leading to better predictions regarding the potential effects of the environment on lineage divergence.

## Introduction

Estimating geographic connectivity among populations has long been of major interest to biogeographers, as ultimately it is the spatial context (and associated ecological factors) that determine the amount of gene flow among lineages (Mayr 1963; Turelli et al. 2001; Mallet et al. 2009; Sobel et al. 2009). Classically, barriers impeding connectivity have been considered to arise as macro‐geographic events (e.g., continental drift, orogenesis, changes in ocean levels, and major river courses; Coyne and Orr 2004; Lomolino et al. 2006; Pyron and Burbrink 2010). The specific isolating mechanisms of such barriers are typically unspecified, but appear to consist of physical/chemical processes that *abruptly* impede dispersal and establishment (e.g., terrestrial species are incapable of regular activity and sustained movement over large bodies of water). More recently, attention has shifted to barriers of an environmental/ecological nature, which influence population demographics without the need of an abrupt physical/chemical barrier (Wiens 2004; McCormack et al. 2010; Glor and Warren 2011; Gutiérrez et al. 2014). In this context, the concept of niche conservatism and its potential pervasiveness has emerged, where populations segregated by unsuitable habitat are posited to remain isolated and potentially diverge due to the tendency to conserve their niches (Wiens 2004; Wiens et al. 2010; Hua and Wiens 2013).

Interest in niche conservatism and environmental barriers has surged with the recent incorporation of GIS‐based tools into biology, in particular correlative ecological niche models (ENMs). Broadly, ENMs rely on the correlation of environmental variables with data documenting species occurrences to estimate species “Grinellian niches” and potential geographic distributions at coarse grains and large extents (reviewed in Peterson et al. 2011). As such, they provide explicit hypotheses of spatial connectivity between populations based on environmental suitability (Wiens and Graham 2005; Kozak et al. 2008; Glor and Warren 2011). For this reason, ENMs have become heavily integrated into the fields of phylogeography and landscape genetics, where they are being used as inputs for powerful simulations aiming to understand the molecular history of lineages (Chan et al. 2011; Alvarado‐Serrano and Knowles 2014).

However, contrary to common perception, violating ENM assumptions is relatively easy, leading to erroneous estimates of niches and geographic distributions that can undermine further analyses (e.g., Lozier et al. 2009; Elith et al. 2010; Anderson 2012, 2013; Araújo and Peterson 2012). Here, we focus on one generally overlooked and potentially common issue: records occurring at spatial margins of species ranges can lead to substantial overestimations of niches, and consequently of the geographic areas that are suitable. This issue was recently explored by Soley‐Guardia et al. (2014), who demonstrated that even if records at spatial margins represent true “sources,” they can inadvertently result in the incorporation of environmental values that typically characterize the surrounding “sink” habitats instead. This can happen when predictor variables lack: (1) accuracy (e.g., insufficient information available during interpolation); (2) sufficient resolution (failing to reflect heterogeneity important to the species), and/or (3) a consistent correlation with relevant proximal variables (i.e., those ultimately determining local suitability *sensu* Austin 2002; see also Anderson 2013). For instance, the higher levels of wetness present within gallery forests might not be detected by precipitation variables, especially if local streams owe their existence to precipitation occurring far beyond. Under these circumstances, variables useful for modeling suitability across most of the species' range, lack the necessary information to discern between conditions allowing persistence along the range margins, and those negating it beyond. This leads to an overestimation of the niche, whence it is inferred that the species can withstand a broader range of environmental conditions than it actually does. The issue is exacerbated at protruding spatially marginal (PSM) localities, where small patches of suitable habitat protrude into otherwise extensive unsuitable regions (Soley‐Guardia et al. 2014).

**Figure 1:**
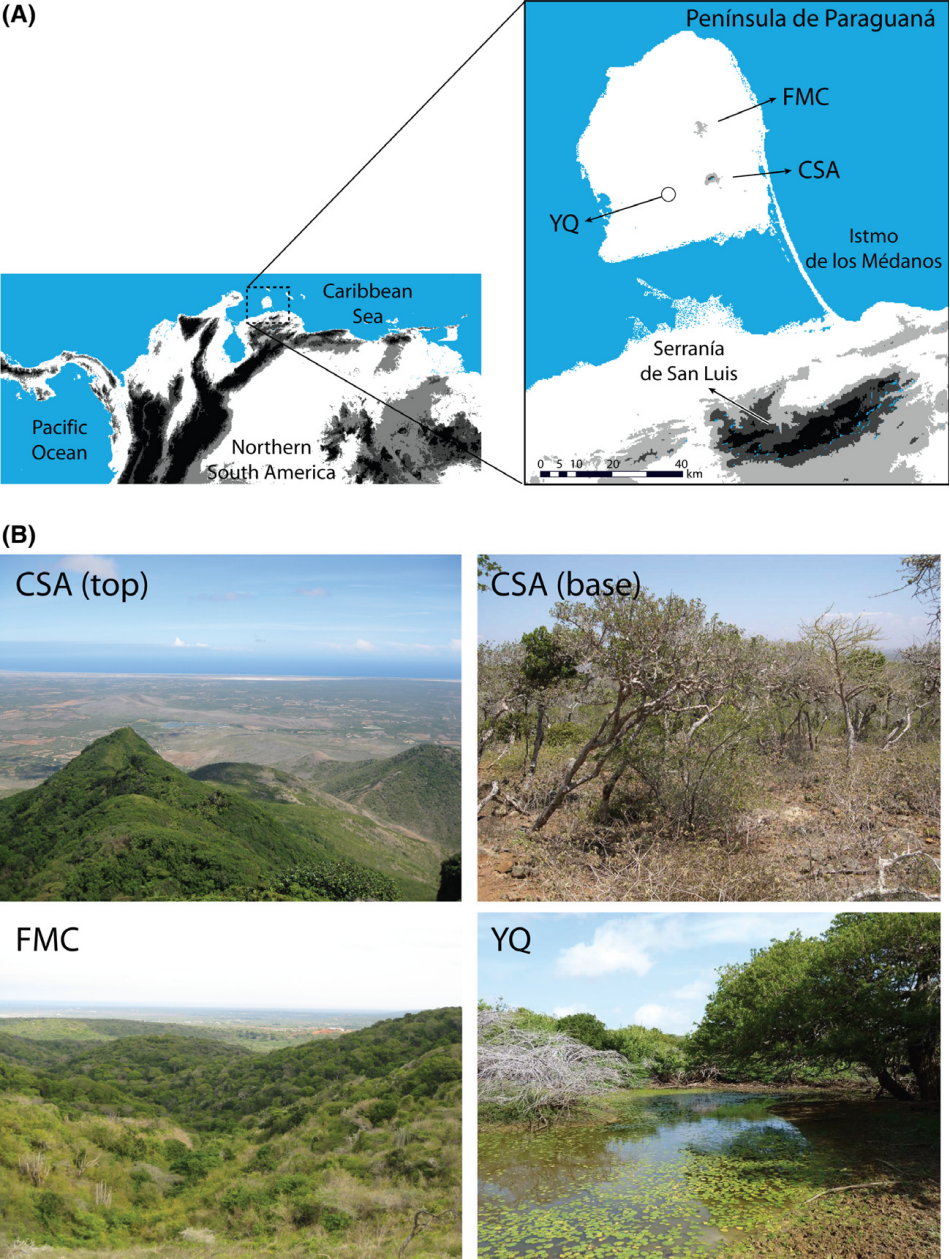
Study system. (A) Digital elevation map showing a close‐up of the Península de Paraguaná in northern South America. Light gray indicates elevations 200–500 m; dark gray 500–1000 m; and black >1000 m. Peninsular sites known to harbor populations of at least one of the study species are shown in bold; CSA: Cerro Santa Ana; FMC: Fila de Monte Cano; YQ: Yabuquiva. (B) Habitat present at, or surrounding, the peninsular sites shown in (A). Mesic habitat on the peninsula is scarce, mostly restricted to Cerro Santa Ana (approximately 850 m in elevation). A few patches of mesic habitat also exist at lower elevations due to local topographic and atmospheric factors. The rest of the peninsula is characterized by xerophytic thorn forests and desert scrub that also extend throughout the narrow isthmus (Istmo de los Médanos; partly exhibiting sand dunes) and adjacent lowlands on the mainland (Markezich et al. 1997; Anderson 2003a; IGVSB 2004; Gutiérrez and Molinari 2008; Anderson et al. 2012). Top pictures: abrupt transition from mesic forests at middle‐to‐high elevations on Cerro Santa Ana, to the xerophytic vegetation predominating in the lowlands (e.g., thorn scrub). Bottom pictures: patches of mesic habitat occurring within the otherwise hot and xeric peninsular lowlands (i.e., protruding spatially marginal localities). Elevation from Shuttle Radar Topography Mission (SRTM), with 3 arc‐second resolution (~90 m), obtained through WeoGeo (http://www.weogeo.com). Photographic credits: CSA top and FMC taken by MSG; CSA base by RPA; YQ by JOG.

The objective of this study was to test whether overestimation of niches due to records occurring at PSM localities can be substantial enough to obscure detection of even stark environmental barriers. We do so in a system consisting of three rodent lineages that inhabit mesic forests in northern South America, including the isolated Península de Paraguaná in northern Venezuela: *Proechimys guairae*,* Rhipidomys venezuelae*, and the species‐pair *Heteromys anomalus*/*H. oasicus*. Mesic habitats on this peninsula are relatively scarce and starkly separated from those on the adjacent mainland by an obvious environmental barrier of hot and xeric lowlands (Fig. 1). Specifically, we built optimally parameterized ENMs for each lineage using only mainland records and projected these models onto the peninsula and intervening lowlands to assess suitability and potential for connectivity among known populations. We predict that the environmental barrier present in this system will be detected only by models built without records from PSM localities (i.e., such records were not present in the occurrence dataset or were subsequently excluded). Additionally, we predict that only a model built including records from PSM localities in the *mainland* will predict as suitable PSM localities in the *peninsula*.

## Materials and Methods

### Study system

The three lineages included in this study consist of the sole rodents known to inhabit mesic conditions within the Península de Paraguaná in northern Venezuela (Anderson et al. 2012). The Guaira Spiny Rat (Echimyidae: *Proechimys guairae*) and the Venezuelan Climbing Mouse (Cricetidae: *Rhipidomys venezuelae*) occur both on the mainland and on Paraguaná (Aguilera et al. 1995; Tribe 1996). The third lineage consists of two closely related species: the Caribbean Spiny Pocket Mouse (Heteromyidae: *Heteromys anomalus*), occurring on the mainland and a few adjacent islands but not on Paraguaná, and its apparent sister species, the Paraguaná Spiny Pocket Mouse (*H. oasicus*), endemic to the peninsula (Anderson 2003a; Anderson et al. 2012; details in Appendix S1).

Together with a mouse opossum (Gutiérrez et al. 2014), these rodents constitute the species‐poor community of small nonvolant mammals occurring in mesic habitat within the peninsula. This community comprises a mere subset of the total diversity of small nonvolant mammals inhabiting mesic conditions within the closest mountain range on the mainland (Serranía de San Luis; 19 species), the diversity of which is, in turn, a subset of that present in other larger mountains of northern Venezuela (Anderson et al. 2012). This nested pattern suggests that hot and xeric lowlands have had an important biogeographic role for mesic species in this region, acting as barriers for dispersal and/or fostering past local extinction (Anderson et al. 2012). Within the peninsula, this notion is supported by the endemicity of several taxa of plants, invertebrates, and vertebrates, including *Heteromys oasicus* (Anderson 2003a; Gutiérrez and Molinari 2008). The nature of such a barrier could have been mostly physical during past time periods (e.g., marine introgressions; Lovejoy et al. 1998; Lara and González 2007). However, we consider that the hot and xeric conditions currently prevailing within the lowlands clearly represent an environmental barrier to mesic‐adapted species today, promoting isolation between mainland and peninsular populations through niche conservatism (Fig. 1).

For the focal lineages, the notion of an environmental barrier is supported by the fact that none of them is known to occur in vast expanses of hot xeric habitat despite intensive sampling for small mammals in northern South America (Handley 1976; Anderson et al. 2012). Similarly, various sampling efforts within hot xeric habitats on the peninsula have failed to detect the focal species there, while successfully detecting other species – for example, *Calomys hummelincki* and *Marmosa xerophila* (Handley 1976; Thielen et al. 2009; Anderson et al. 2012; see also Rossi et al. 2010; Gutiérrez et al. 2014). Given that mammalian surveys use fairly standardized methods for sampling (Wilson et al. 1996), the possibility of artifactual absences is unlikely (Anderson 2003b; Phillips et al. 2009; Yackulic et al. 2013). Therefore, rather than testing the hypothesis that the hot and xeric lowlands act as an environmental barrier, we consider this a reasonable assumption in the present system. Instead, we ask whether the ability of ENMs to detect this barrier using readily available and typically useful environmental variables (i.e., WorldClim bioclimatic layers) is affected by the presence of PSM localities.

### Testing the effect of PSM localities

To test the effect of PSM localities on the ability of ENMs to detect the environmental barrier, we relied on a simple yet intuitive approach. First, for each lineage, we built optimally parameterized models using *mainland* records only and projected them onto the peninsula and intervening lowlands. Then, we investigated whether records from the mainland receiving low suitability values represented PSM localities – and if so, we built and projected a second model without using these records. As it is also possible that PSM localities exist in the peninsula, we explored whether *peninsular* records corresponded to PSM localities as well. Whereas other approaches for detecting environmental barriers have been proposed (e.g., reciprocal modeling and prediction; Warren et al. 2008, 2010), we implemented this particular one given that several realities precluded the building of sensical ENMs for peninsular populations (i.e., few spatially independent records, a very small accessible area, and narrow range of environmental conditions present; Appendix S1).

We predict that the environmental barrier present in the projection region (i.e., isthmus and adjacent peninsular lowlands) will only be detected by the ENMs that were built without records from PSM localities. Additionally, we predict that if *peninsular* PSM localities exist, they will only be predicted as suitable by ENMs calibrated with datasets including records from *mainland* PSM localities. To test these predictions, we transformed the continuous outputs of the ENMs into categorical ones by applying two thresholds (based on suitability values assigned to particular mainland records; see Appendix S1 for details and continuous outputs). The first one indicates all areas suitable to the species (“lenient threshold”), whereas the second one demarcates areas of higher suitability (“stricter threshold”). In cases where records from PSM localities were found to have been included in the original model, this second threshold restricts suitability to areas receiving higher values than those records (to assess whether that approach proofed sufficient to counter their effect).

Finally, as neither the original nor the second models are fully correct on their own when PSM localities are present in the calibration data (i.e., the first one suffers from commission and the second one from omission), we made a composite prediction following Soley‐Guardia et al. (2014). To do so, we overlaid the categorical estimate of suitability of the model built without records from PSM localities (using both thresholds) on top of the binary estimate of suitability of the model built with all records (using the lenient threshold). This composite prediction distinguishes between areas harboring the typical conditions inhabited by the species vs. areas that are typically unsuitable but where the species might occur locally *if* the necessary factors are present (considered suitable only by the model built with all records; Soley‐Guardia et al. 2014). Areas not considered as suitable by either model at the lenient threshold are deemed unsuitable.

### Ecological niche modeling and detecting protruding spatially marginal localities

We obtained occurrence records from the literature and our fieldwork, representing specimens verified by experts and georeferenced carefully (Appendix S1). Aiming to reduce the potential for biased niche inferences stemming from sampling biases (Hortal et al. 2008; Merow et al. 2013), we spatially filtered (thinned) mainland records (Kramer‐Schadt et al. 2013; Syfert et al. 2013; Boria et al. 2014). This yielded 56 mainland records for *Proechimys guairae*, and 22 for *Rhipidomys venezuelae* (Appendix S1). For *Heteromys anomalus*, we used the dataset of Soley‐Guardia et al. (2014), consisting of 126 records. Peninsular records (six for *P. guairae*, six for *R. venezuelae*, and seven for *H. oasicus*) were not used during calibration; hence, they were not filtered, leaving all of them as tests of the models. We delimited calibration regions following principles of Anderson and Raza (2010) to reduce the likelihood of violating sampling, dispersal‐related, and biotic assumptions (see also Barve et al. 2011; Saupe et al. 2012; Anderson 2013). Calibration regions corresponded to a rectangle encompassing all records after filtering, having the following coordinates for *P. guairae*,* R. venezuelae*, and *H. anomalus*, respectively: 8.00–11.50° N, 63.50–72.00° W; 8.00–11.50° N, 66.50–74.50° W; and 7.50–11.50° N, 60.00–77.00° W. For all three lineages, the region to which models were projected had the same coordinates as the calibration region, except for the northern limit, which was extended to 13.00° N to include the isthmus and peninsula.

We built models for each species in maxent 3.3.1 (Phillips et al. 2006), an ENM algorithm that has been widely used to infer environmental effects on lineage divergence (e.g., Kozak and Wiens 2006; Warren et al. 2010; Glor and Warren 2011). As *potential* predictors, we used the bioclimatic variables from WorldClim, which have a resolution of ca. 1 km^2^ at the equator (Hijmans et al. 2005). Given that our modeling goals are predictive rather than explanatory (i.e., predicting suitability rather than elucidating driving variables), we used the complete set of 19 variables under a machine‐learning approach (Breiman 2001; Araújo and Guisan 2006; Olden et al. 2008; Elith et al. 2011). However, to approximate optimal model dimensionality (number of variables *actually* incorporated into the model) and complexity (parameters modeling the response to each variable *incorporated* into the model; Merow et al. 2013), we evaluated the predictive performance of preliminary models using spatially independent splits of the data (Appendix S1; Wenger and Olden 2012; Radosavljevic and Anderson 2014).

For each species, after determining the settings that yielded models with the highest average predictive performance, we built a final model using those settings and all mainland records. This optimally parameterized model was projected onto the peninsula. For each lineage, the same settings were also used later for the model built without records from PSM localities in order to facilitate comparisons. To assess whether estimates of suitability within the projection region could be hampered by the presence of nonanalog environments there (Williams and Jackson 2007), we inspected the “multivariate environmental similarity surface” (MESS) and “most dissimilar variable” (MoD) figures produced by maxent (Elith et al. 2010) and compared them with modeled response curves (Anderson 2013). This procedure revealed that peninsular environments almost completely fall within the range of conditions of model calibration (i.e., little need for model extrapolation), and therefore that estimates of suitability there are not affected by the two different ways in which maxent deals with extrapolation (i.e., clamping vs. not; Appendix S1).

To identify PSM localities, we follow the approach proposed by Soley‐Guardia et al. (2014), who developed it using one of our study species, *Heteromys anomalus*. This consisted of retrieving habitat descriptions only for the set of mainland records given the lowest suitability by the optimally parameterized model. Ideally, habitat descriptions could be retrieved for every record to determine whether they represent PSM localities; however, such a procedure would be unnecessarily laborious and time‐consuming. The premise behind the procedure followed here is that if records from PSM localities misinform the model, they will be given a lower suitability than other records. If records from PSM localities receive a high suitability, they evidently do not suffer from the issues mentioned in the introduction (or they represent the majority of records, in which case the researcher should reconsider the modeling exercise all together). Specifically, we ranked each species records according to the suitability values they received in the optimally parameterized model and plotted these against the suitability values themselves to detect strings of records receiving particularly low scores (i.e., separated by a strong gap from the rest). Analyzing a large number of records for *H. anomalus*, Soley‐Guardia et al. (2014) found that most records associated with PSM localities corresponded to those below the lowest gap in suitability. Hence, we decided to obtain habitat descriptions only for the records spanning the two lowest gaps in suitability. We did so using published literature, field notes, communication with collectors, and regional vegetation maps (IIRBAVH 1998; IGAC 2003; IGVSB 2004). We considered records to represent PSM localities only if the habitat associated with them corresponded to *natural* vegetation mosaics, where the species' typical mesic habitat is intermixed with habitats characterizing hotter and drier regions (e.g., gallery forests within thorn scrub or natural savannas). “Mosaics” resulting from anthropogenic deforestation were not considered as PSM localities, as areas holding such artificial mosaics are still characterized by the same meteorological phenomena that resulted in the original forests, and which should be correctly represented by the climatic variables used in this study. Finally, we examined habitat descriptions for all peninsular records, as these represented a small number and were crucial for interpreting the models.

## Results

### Detection and exclusion of records from protruding spatially marginal localities

For all species, the suitability vs. rank plots revealed substantial gaps among the lowest‐ranking localities, potentially indicating major changes in environmental characterization. Specifically, the four lowest‐ranking records in *Proechimys guairae*, and five in *Rhipidomys venezuelae*, spanned two substantial gaps in suitability (Appendix S1). Therefore, we gathered habitat descriptions for the five lowest‐ranking records in both species. For *P. guairae*, all five corresponded to localities originally characterized by extensive semi‐deciduous or deciduous forests (i.e., no records from PSM localities; Appendix S1). For this reason, we consider that the low suitability of these records is real rather than an artifactual result from issues of spatial marginality (Soley‐Guardia et al. 2014). Consequently, we did not build a second model for this species. For *R. venezuelae*, we detected two records occurring at PSM localities, which corresponded to those assigned the lowest ranks. These records represented instances where the species was collected within locally mesic conditions existing within otherwise hot dry regions (PSM localities). The three other records analyzed for this species consisted of captures within extensive evergreen forests (Appendix S1). Therefore, for this species, we excluded the two lowest‐ranking records from the calibration of the second model. For *Heteromys anomalus*, Soley‐Guardia et al. (2014) found that the 15 lowest‐ranking records corresponded to PSM localities, where the individuals were collected mostly in gallery forests surrounded either by xerophytic thorn scrub or natural savannas. The higher‐ranking records for which those authors obtained information corresponded mostly to captures within evergreen and deciduous forests. Hence, we excluded the 15 lowest‐ranking *H. anomalus* records from the second model. Regarding peninsular records, habitat descriptions led us to consider all but those from Cerro Santa Ana as representing PSM localities (the locality of Moruy, near the base of this mountain, also represents a PSM locality). Importantly, whenever sufficiently detailed information was available (whether in the mainland or the peninsula), it revealed that at PSM localities, specimens were always collected within the mesic patches or close by in one instance (rather than in the widely available hot and xeric habitats; Appendix S1; see also Soley‐Guardia et al. 2014).

### Detecting the environmental barrier and estimating peninsular suitability

#### Interpretations under the lenient threshold

As expected, PSM localities had a major effect on estimates of suitability. Overall, models calibrated with records from PSM localities were substantially more expansive. Within the mainland, in addition to the mesic regions characterized by the typical habitat of the species, these models also considered as suitable extensive regions of hot arid and semi‐arid lowlands (e.g., coastal areas of northern South America, the *llanos*), characterized by habitats where these species do not persist (e.g., xerophytic thorn forests, desert scrub, and grassland savannas). In contrast, models calibrated without these records were more realistic, restricting suitability to mesic regions only. However, as expected, the latter models naturally resulted in omission of the records from PSM localities that were not used during calibration (see Appendix S1 for estimates of suitability across the entire study region).

**Figure 2:**
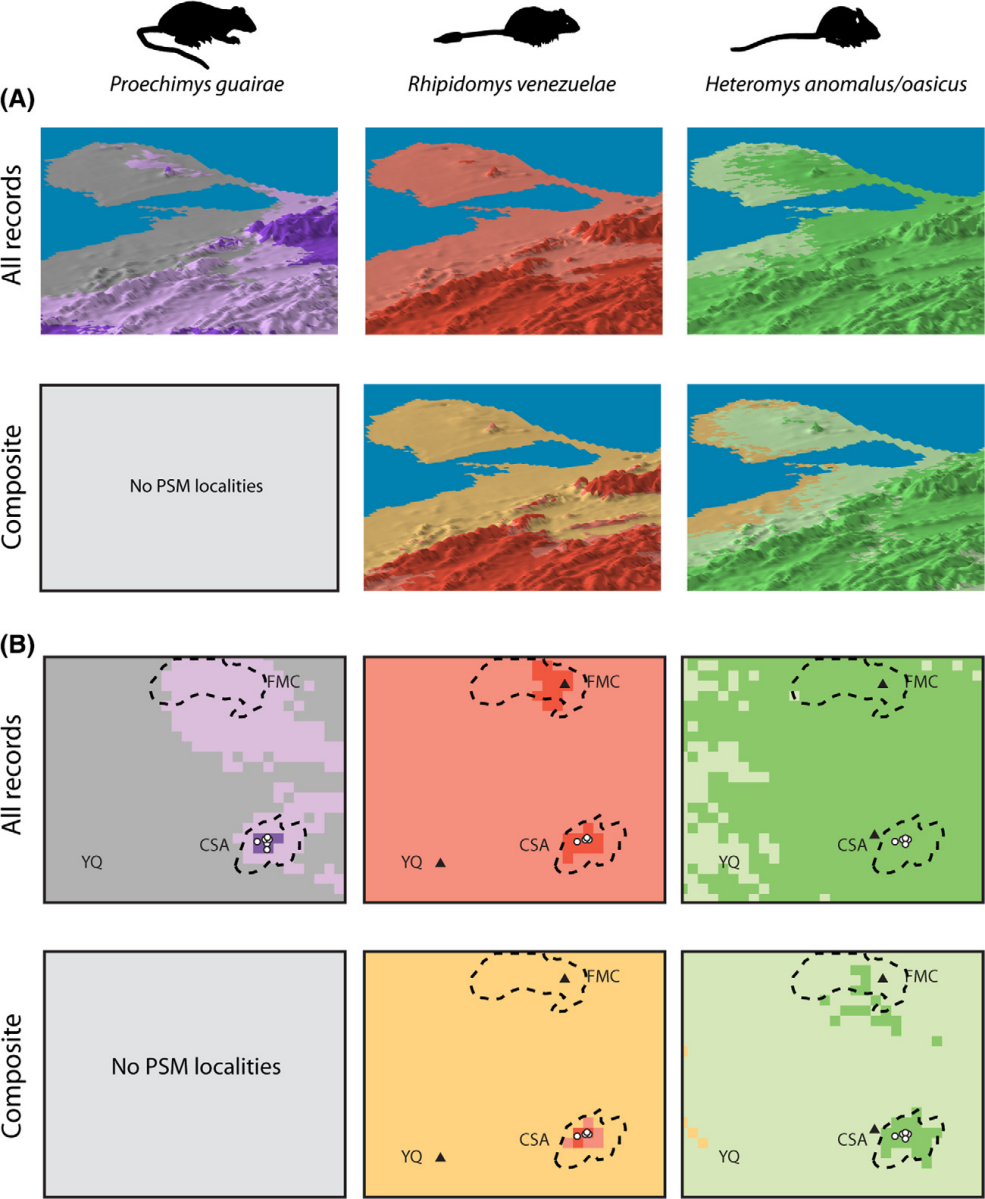
Projections of maxent models onto the Península de Paraguaná in northern South America, showing categorical estimates of suitability for each lineage. Predictions correspond to either models built with all records, or to composite predictions – the latter made by overlaying the categorical estimates of suitability obtained from the models built without records from protruding spatially marginal (PSM) localities, on top of the binary estimates of suitability obtained from the models built with all records. Gray: unsuitable areas; pale colors: areas of low suitability (suitable only at the lenient threshold); dark colors: areas of higher suitability (suitable at both the lenient and the species‐specific stricter thresholds; details in text). In the composite predictions, the tan color indicates areas suitable only in the models built with all records and at the lenient threshold (denoting areas where the species might occur *if* locally mesic conditions exist). (A) Suitability draped over an elevation surface (the latter exaggerated for clarity). Shading according to elevation is provided for visual purposes and does not constitute a color gradient. Note differences in the potential for geographic connectivity among mainland and peninsular populations according to the different models. (B) Close‐up of the projections shown in (A) – within the center of the peninsula. Each pixel measures ~1 km^2^. Symbols indicate known peninsular records of the studied species, with triangles marking those occurring within PSM localities. Dashed lines indicate approximate contours of areas of higher elevation (*ca*. 150 m) within the peninsula. CSA: Cerro Santa Ana; FMC: Fila de Monte Cano; YQ: Yabuquiva. Note qualitative differences in suitability assigned to PSM localities (and areas between them) by the different models. Projections were made in arcscene ^®^ 9.2 (ESRI, Redlands, CA, USA). Elevation from Shuttle Radar Topography Mission (SRTM), with 3 arc‐second resolution (~90 m), obtained through WeoGeo (http://www.weogeo.com).

Most importantly, the effect of PSM localities was substantial enough as to yield models that did not detect the environmental barrier present in this system. In general, the effect of PSM localities within the projection region matched our experimental predictions. For *Proechimys guairae*, the sole species where none of the examined records represented PSM localities (neither on the mainland nor the peninsula), the model built with all records correctly identified the environmental barrier of the isthmus and adjacent peninsular lowlands (Fig. 2A). Additionally, this model restricted peninsular suitability almost exclusively to the mesic habitats of Cerro Santa Ana. However, contrary to predictions, the model also considered as suitable the peninsular PSM locality of Fila de Monte Cano (despite being calibrated without records from PSM localities in the mainland). The fact that no records of this species exist there is of less relevance to this study (i.e., it could represent biases in detection and dispersal, rather than a true commission error).

For *Rhipidomys venezuelae*, estimates of suitability within the projection region differed dramatically between models made including or excluding mainland records from PSM localities. The environmental barrier of the isthmus and adjacent lowlands was correctly detected only by the model built without the two records from PSM localities (Fig. 2A). Also as predicted, this model restricted peninsular suitability exclusively to the mesic Cerro Santa Ana (Fig. 2B). In turn, the model built with all records considered as suitable the entirety of the peninsula, including the PSM localities there.

For the *Heteromys* lineage, neither model was able to detect the environmental barrier of the isthmus and adjacent lowlands, implying potential for connectivity between the two species of spiny pocket mice (Fig. 2A). The model built with all records considered the entirety of the peninsula as suitable, whereas the one built excluding records from PSM localities was almost as permissive, only considering as unsuitable the peninsular coastal areas (but see *interpretations under the stricter threshold* below). In this way, both models considered as suitable the mesic Cerro Santa Ana, as well as the peninsular PSM localities (Fig. 2B).

#### Interpretations under the stricter threshold

The possibility of countering the effect of PSM localities simply using a stricter threshold in the models built with all records showed ambiguous results. In the case of *Rhipidomys venezuelae*, use of the stricter threshold in the model built with all records did result in detection of the environmental barrier (Fig. 2A). This threshold also considered as unsuitable most peninsular PSM localities, with the exception of Fila de Monte Cano (Fig. 2B). In contrast, for the *Heteromys* lineage, use of the stricter threshold in the model built with all records did not lead to detection of the environmental barrier (Fig. 2A). Similarly, most of the peninsula was still considered suitable under this threshold, including the peninsular PSM localities (Fig. 2B).

In the models built *without* records from PSM localities, use of a stricter threshold did help depict a clearer picture regarding peninsular suitability for all lineages (Fig. 2B). For *Proechimys guairae*, the stricter threshold still considered the mesic Cerro Santa Ana as suitable, but not the PSM locality of Fila de Monte Cano (considered suitable under the lenient threshold). Similarly, for *Rhipidomys venezuelae*, use of the stricter threshold in the model built without records from PSM localities restricted suitability to the higher areas of Cerro Santa Ana, corresponding to the areas where this species has been captured there. For the *Heteromys* lineage, use of the stricter threshold in the model built without records from PSM localities resulted in detection of the environmental barrier. Additionally, this threshold restricted suitability almost exclusively to Cerro Santa Ana (part of the PSM locality of Fila de Monte Cano was also deemed suitable).

## Discussion

### The effects of protruding spatially marginal localities

The results of this study demonstrate that records from PSM localities can lead to ENMs that overestimate species niches, and consequently the extent of their potential geographic ranges (i.e., abiotically suitable areas of Peterson et al. 2011, p. 31). Most importantly, as was evidenced for *Rhipidomys venezuelae*, this pernicious effect can be triggered by only a few such records. Here, we were specifically interested in the effect that PSM localities could have in the detection of an obvious environmental barrier. This barrier was easily detected in the lineage that did not present records at PSM localities, *Proechimys guairae*. However, detection of the environmental barrier in the two lineages presenting records at PSM localities required accounting for their effects in the models. For *R. venezuelae*, exclusion of the two records representing PSM localities when building the model was sufficient. However, detection of the barrier in *Heteromys* required use of a stricter suitability threshold, in addition to exclusion of records from PSM localities. These procedures also led to more realistic inferences within the rest of the peninsula, where high suitability was assigned exclusively to the mesic Cerro Santa Ana. These inferences are in line with what is currently known for this system, and they suggest that unless researchers are familiar with their systems, records from PSM localities can lead to erroneous conclusions.

Differences in ENMs built including vs. excluding records from PSM localities result from the environmental information that such records provide and do not constitute a mere sample‐size effect (Soley‐Guardia et al. 2014). The environments corresponding to records from PSM localities differ from those corresponding to the rest of records (Table 1). This environmental difference is also evident in both the low prediction values that records from PSM localities received in the original model, and the extensive hot and dry areas that were *only* predicted as suitable in that model (Figs. 2A; S1). Even though patches of mesic habitat (i.e., PSM localities) can occasionally occur within these hot and dry areas, this is not typically the case within the study region. Instead, hot and dry conditions usually define vast expanses of xerophytic vegetation (IGAC 2003; IGVSB 2004), habitats that the focal species are not known to occupy. Not surprisingly then, including records from PSM localities to calibrate models resulted in predictions that indicated as suitable what is really an environmental barrier of hot and xeric habitats (Fig. 2A).

**Table 1:** Environmental values (means and ranges) for different sets of occurrence records of each of the three lineages studied. The two variables included herein correspond to those with high “percent contribution” during internal iterations of the generation of each MaxEnt model. Because of possible differences in environmental signals between occurrence datasets, as well as the machine‐learning approach used by MaxEnt, the identity of the two variables with the highest importance differed for each specific model (i.e., calibrated with vs. without records from protruding spatially marginal (PSM) localities). For presentation, we chose variables that had a high “percent contribution” in both models (percentages shown in parentheses), and which were also included in each respective final model (i.e., present with nonzero weights in the “lambdas” file). Conveniently, these corresponded to both temperature (°C) and precipitation (mm) variables for each lineage. For *Heteromys anomalus*, the “mainland regular localities” consist mostly of extensive forests; however, that dataset also includes four PSM localities that received higher rankings, and seven localities for which no habitat descriptions were found (Soley‐Guardia et al. 2014). *n*: Sample size for each successive column from left to right; NA: not applicable (i.e., no PSM localities were found). Although no statistical tests are conducted here, note that for *Rhipidomys venezuelae* and the *Heteromys* lineage, PSM localities unambiguously showed substantially higher means for temperature and markedly lower means for precipitation (for both mainland and peninsular comparisons)

Lineage	Variables: % contribution (All records/ Excluding PSM localities)	Mainland PSM localities (vegetation mosaics)	Mainland regular localities (extensive forests)	Mainland rest of localities (highest suitability values; not inspected)	Peninsular PSM localities (vegetation mosaics)	Peninsular regular localities (extensive forests)
*Proechimys guairae n *=* *0; 5; 51; 0; 6	Temperature annual range (16%/NA)	NA	13 (11–14)	13 (9–16)	NA	12 (12–12)
	Precipitation of driest quarter (23%/NA)	NA	37 (25–51)	86 (16–253)	NA	73 (59–76)
*Rhipidomys venezuelae n *=* *2; 3; 17; 2; 4	Maximum temperature of warmest month (76/63%)	35 (34–36)	32 (31–32)	26 (21–31)	33 (32–34)	30 (30–30)
	Precipitation of driest quarter (5/2%)	37 (13–61)	125 (60–200)	87 (32–141)	44 (31–56)	76 (75–76)
*Heteromys anomalus/oasicus n *=* *15; 39; 72; 2; 5	Maximum temperature of warmest month (44/41%)	34 (31–36)	32 (22–36)	29 (20–34)	33 (32–33)	30 (30–30)
	Precipitation of driest quarter (9/15%)	38 (7–152)	92 (12–276)	144 (35–322)	51 (46–56)	76 (75–76)

### Composite predictions improve and enrich inferences

Calibrating ENMs with records found at PSM localities results in inflated estimates of suitability (i.e., commission errors); however, removing them altogether inherently underestimates the regions suitable to a species (i.e., omission errors). The alternative of choosing stricter thresholds to define suitability conceptually suffers from the same issue. Such a procedure might alleviate the effect of PSM localities in some instances, as was evidenced for *Rhipidomys venezuelae*. However, a priori knowledge regarding the fraction of records representing PSM localities would be needed to give the threshold a straightforward interpretation (i.e., denotes areas that are occasionally suitable), and even then, the interpretation will not be as direct (i.e., the niche was still inflated during calibration; Soley‐Guardia et al. 2014).

Instead, jointly interpreting models built including vs. excluding records from PSM localities better deals with the issue presented by these records, providing a richer product with more straightforward interpretations (and additionally, not suffering from systematic omission or commission errors; Soley‐Guardia et al. 2014). For instance, in this system, the composite prediction for *Rhipidomys venezuelae* revealed the existence of the environmental barrier. However, it also recognized that this barrier is characterized by environmental conditions that can occasionally hold locally suitable mesic habitat if appropriate factors are present (Fig. 2A). This is the case for the peninsular PSM localities of Fila de Monte Cano and Yabuquiva, which do harbor records of this species. These localities are recognized as suitable in the composite prediction under the special category of “*as long as”* necessary local factors creating mesic conditions are present (Fig. 2B). In this way, even though the hot and xeric lowlands typically act as an environmental barrier, such a barrier might occasionally be breached if the necessary local factors are present long enough, creating pockets of PSM localities that can be used as stepping stones – that is, resulting in “soft allopatry” (see Fransen 2002 and Gutiérrez et al. 2014 for common use of “soft vicariance” to imply incomplete isolation regardless of a barrier's nature).

As follows, accounting for the effect of PSM localities seems especially relevant for studies that integrate ENMs with molecular analyses to elucidate the role of past environmental changes on lineage divergence and genetic structuring (e.g., Waltari et al. 2007; Carnaval et al. 2009; Chan et al. 2011; Alvarado‐Serrano and Knowles 2014). Given the substantial advancements in that field, researchers currently aim for ever‐more detailed reconstructions of the conditions under which particular lineages diverged (Knowles and Maddison 2002; Hickerson et al. 2010). For instance, genetic correspondence with porous barriers identified through procedures similar to the present study might serve as strong support for an “isolation with migration” model (Hey 2010).

Additionally, composite predictions can provide further insight into potential evolutionary processes acting within a region. In this system, it is possible that the small areal extents of PSM localities coupled with their proximity to unsuitable environments probably result in different conditions than those typically experienced by the species. This seems likely given the vegetational composition of mesic patches at such localities, which typically include some xerophytic vegetational elements (Fig. 1; Appendix S1). These conditions might make PSM localities less suitable or even environmentally marginal to the species (i.e., barely allowing population growth; see Soley‐Guardia et al. 2014 for the distinction between *spatial* and *environmental* marginality). In this way, populations at PSM localities might experience different selection pressures, potentially promoting niche evolution on behavioral or physiological axes. Along these lines, of the lineages included in this study, *Proechimys guairae* seems to have the most restrictive mesic niche. In contrast, *Rhipidomys venezuelae* and the *Heteromys* lineage apparently have less‐restrictive mesic niches that allow them to inhabit extensive evergreen and deciduous forests, as well as heterogeneous mosaics where these forests mix with xerophytic elements. Although both lineages occur in mosaics on the mainland and the peninsula, the potential for local adaptation in the latter seems more likely given the spatial isolation there (i.e., avoiding “genetic swamping” by migrants from populations inhabiting extensive optimal habitat; Bridle and Vines 2007; Kawecki 2008). The isolation of peninsular PSM localities is mostly or only evident in the composite predictions (Fig. 2).

### Idiosyncratic effects of protruding spatially marginal localities

The precise effect of records from PSM localities in any ENM will depend upon the idiosyncrasies of each dataset. Firstly, different PSM localities likely differ in the degree to which they are affected by issues related to spatial marginality (e.g., regarding how accurately their environments are represented by the variables used). Secondly, the effect of records from PSM localities takes place within the environmental context represented by the totality of occurrence records used to calibrate the model. For instance, average environmental values of occurrence records (or their range of variation) can constitute constraints that a maxent model aims to satisfy (Merow et al. 2013). In this way, a particular record from a PSM locality can have different effects in various occurrence datasets. Thirdly, the effect of a particular record will also depend upon the environmental space represented by the sample against which occurrences are contrasted. In maxent, occurrences are contrasted against a background sample (i.e., environments *available* to the species), and the effect of a particular PSM locality will likely be stronger when the environments it represents are uncommon in such a sample (Merow et al. 2013).

Finally, it is important to note that under a machine‐learning approach such as maxent, the environmental characterization of records is dependent not only upon the variables and constraints allowed by the user, but also upon whether these prove informative during calibration (Breiman 2001; Olden et al. 2008). In this way, the exact effect of any one record from a PSM locality can be contingent upon model parameterization, opening the possibility that records from PSM localities not affecting the first model (and consequently given a high rank and remaining undetected), might affect the second one. Factors related to such an issue could have been responsible for the still unrealistic prediction of the second model built for *Heteromys anomalus*, in contrast with the realistic prediction obtained for *Rhipidomys venezuelae* (Appendix S1).

### Conclusions and future directions

In this study, PSM localities obscured the detection of a stark environmental barrier. Without proper consideration of this issue, the effect of niche conservatism as an agent driving allopatry and divergence (Wiens 2004; Hua and Wiens 2013) could erroneously be ruled out, leading researchers to propose alternative hypotheses. For instance, in the case of *Heteromys anomalus*, uncritically accepting the model built with all records would beg for additional explanations as to why this species does not currently extend its distribution into the lowlands of the peninsula, or even into the range of *H. oasicus* (e.g., competition). Alternatively, if PSM localities had been represented in only one of the datasets for a given lineage (i.e., mainland vs. peninsula), incorrect conclusions about niche evolution (e.g., contraction or expansion) could have been reached. In particular, this latter possibility represents a potential caveat for tools commonly applied to compare niches either in geographic or environmental space (e.g., McCormack et al. 2010; Warren et al. 2010; Broennimann et al. 2012), regardless of whether they use outputs from ENMs or are based on direct comparisons of environmental data.

The adverse effect of PSM localities is caused by the (occasionally) inconsistent correlation of environmental variables with suitable and unsuitable habitat. In this sense, there is great potential for remotely sensed variables to ameliorate this issue by providing variables with fine resolution (e.g., vegetation indices) that are more tightly correlated with proximal factors relevant to the species. However, substantial development is still needed in this area regarding data availability, transformation, and interpretation (Shirley et al. 2013). Moreover, such data will typically be unavailable for past or future time periods.

In the meantime, procedures similar to the one implemented in this study can be useful when researchers suspect the existence of PSM localities in their datasets. In our case, a rapid inspection of suitability plots plus careful gathering of habitat descriptions for a subset of records led to the discovery of important PSM localities. Then, a joint interpretation of the models built including vs. excluding records from these localities led to more realistic inferences according to what is known for this system. In this way, rather than uncritically accepting outputs from ENMs and associated tools, researchers can be encouraged to leverage such outputs with available natural history information, carefully assessing whether results are biologically realistic.

## Conflict of Interest

None declared.

## Supporting information


**Appendix S1.** Methodological details and additional results.
**Table S1.** Average evaluation scores of preliminary ecological niche models for *Proechimys guairae* calibrated in maxent with various settings.
**Table S2.** Average evaluation scores of preliminary ecological niche models for *Rhipidomys venezuelae* calibrated in maxent with various settings.
**Table S3.** Details of the final ecological niche models calibrated for each species using settings deemed as optimal in the preliminary models.
**Table S4.** Habitat information used to determine whether inspected records corresponded to protruding spatially marginal (PSM) localities.
**Table S5.** Thresholds used in each species to transform the continuous estimates of suitability of the maxent models into categorical ones.
**Figure S1.** Plot used to identify the least‐suitable records on the mainland, several of which represented protruding spatially marginal (PSM) localities in two of the three lineages.
**Figure S2.** Estimates of suitability across the entire study region for each lineage, according to ecological niche models built including and excluding records occurring at protruding spatially marginal (PSM) localities.
