# Supplementary material for: Are we overestimating the niche? Removing marginal localities helps ecological niche models detect environmental barriers
Source: Ecol Evol. 2016 Jan 28;6(5):1267–79. doi: 10.1002/ece3.1900 (PMC4730904; doi:10.1002/ece3.1900)
Supplement: Supplementary file 1 — Appendix S1. Methodological details and additional results. Table S1. Average evaluation scores of preliminary ecological niche models for Proechimys guairae calibrated in maxent with various settings. Table S2. Average evaluation scores of preliminary ecological niche models for Rhipidomys venezuelae calibrated in maxent with various settings. Table S3. Details of the final ecological niche models calibrated for each species using settings deemed as optimal in the preliminary models. Table S4. Habitat information used to determine whether inspected records corresponded to protruding spatially marginal (PSM) localities. Table S5. Thresholds used in each species to transform the continuous estimates of suitability of the maxent models into categorical ones. Figure S1. Plot used to identify the least‐suitable records on the mainland, several of which represented protruding spatially marginal (PSM) localities in two of the three lineages. Figure S2. Estimates of suitability across the entire study region for each lineage, according to ecological niche models built including and excluding records occurring at protruding spatially marginal (PSM) localities. [file ECE3-6-1267-s001.pdf]

Appendix S1

Methodological details and additional results

Experimental design

Several approaches using ENMs have been proposed to test for niche conservatism and its importance in fostering isolation between populations (e.g., Kozak and Wiens 2006; Warren et al. 2008, 2010; Broennimann et al. 2012). These rely on building models for each set of populations, which in our case was not feasible, given that peninsular populations are restricted to very few sites on Paraguaná (1–3 independent localities), with most records being tightly clustered on Cerro Santa Ana (Fig. 1 of main text). This biological reality, together with the small extent and geographic isolation of the peninsula (yielding a very small accessible area and narrow range of environmental conditions), precludes the building of appropriate ENMs for peninsular populations (Peterson et al. 2011, pp. 29, 40; Saupe et al. 2012).

Data gathering and processing

The rodent lineages included in this study typically occur in mesic habitats from ca. 0–1600 m (and occasionally higher for *Rhipidomys venezuelae* and *Heteromys anomalus*) throughout northern South America (Handley 1976; Tribe 1996; Anderson 2003; Anderson and Gutiérrez 2009; Anderson et al. 2012). *Proechimys guairae* occurs only on continental Venezuela, whereas *R. venezuelae* also ranges onto continental Colombia (Aguilera et al. 1995; Tribe 1996). *Heteromys anomalus* occurs on continental Colombia and Venezuela, as well as the islands of

Margarita, Trinidad, and Tobago. Its likely sister species, *H. oasicus*, occurs only on the Península de Paraguaná, Venezuela, on which *H. anomalus* is absent (Anderson 2003; Anderson and Gutiérrez 2009).

We obtained occurrence records throughout the range of these species from the literature and our own fieldwork. For *Proechimys guairae*, we included records from a karyological analysis of Venezuelan spiny rat species (Aguilera et al. 1995). We used all records of *P. guairae* reported in that study, except those corresponding to "*P. g.* Barinas subsp." (currently not recognized as part of this species; see Carleton and Musser 2005). Additionally, we included records formerly referred to as *P. semispinosus* (from Handley 1976) that according to current taxonomy correspond to *P. guairae*—see correspondence between provenance of localities of these records and the geographic range of *P. guairae* described in the karyological study of Aguilera et al. (1995). Finally, we also added records from our fieldwork in northern Venezuela (Anderson et al. 2012). For *Rhipidomys venezuelae*, we obtained the majority of our records from the most recent taxonomic revision of the genus (Tribe 1996). In that revision, *R. venezuelae* was treated as "*R. latimanus venezuelae*", but later this taxon was reinstated to species level (Voss et al. 2001). This occurrence dataset was supplemented by records from our fieldwork as well (Anderson et al. 2012). For *Heteromys anomalus,* we used data from Soley-Guardia et al. (2014), who obtained occurrence records from Anderson (2003), Anderson and Gutiérrez (2009), and Anderson et al. (2012). We obtained records of *H. oasicus* from our field work, as well as from an exhaustive revision of museum specimens (Anderson 2003 and additional localities reported here). Peninsular collecting localities not described in any of the aforementioned studies are documented in the gazetteer at the end of this document.

We obtained geographic coordinates for these records following several steps. For many records, coordinates of collecting localities had already been published following extensive georeferencing efforts (e.g., Paynter 1982, 1997; Anderson et al. 2012; Soley-Guardia et al. 2014), and we used those in the present study. However, for records lacking this information (or where published coordinates were suspected of large error), we obtained geographic coordinates using detailed 1:100,000 and 1:25,000 state maps, according to site descriptions on specimen tags, collecting catalogs, and/or field notes. Estimates of potential georeferencing error are not documented in several of the published sources; however, where documented, it is estimated to be < 5 km for most localities, but up to 10 km for some (e.g., Anderson et al. 2012). We estimate that most georeferences obtained in the preset study have a maximum potential error of < 5 km, but this was not explicitly calculated in all instances.

Aiming to reduce the effect of sampling biases in niche inferences (Hortal et al. 2008; Phillips et al. 2009), we spatially filtered the occurrence records (Kramer-Schadt et al. 2013; Syfert et al. 2013; Boria et al. 2014). Specifically, we followed the approach implemented by Anderson and Raza (2010), and retained the maximum number of records that were separated from each other by an Euclidean distance of at least 10 km. For each cluster of records, we measured distances in ARCGIS® 9.2 (ESRI, Redlands, CA, USA), and assessed all possible solutions. If multiple co-optimal solutions were possible, we randomly chose one, or gave priority to those including records with GPS coordinates when possible. These resulted in a total of 56 records for *Proechimys guairae* (from 83 original unfiltered records), and 22 for *Rhipidomys venezuelae* (from 34 original unfiltered records). The spatially filtered data compiled for *Heteromys anomalus* by Soley-Guardia et al. (2014) consisted of 126 records (from 215 original unfiltered records).

Study regions used to calibrate the models were demarcated by a rectangle encompassing all records after filtering, which was delimited by the nearest even 0.5° that was at least 20 km away from the most peripheral record in each cardinal direction (exact coordinates given in the main text). This delimitation scheme was chosen to reduce the likelihood of violating modeling assumptions (Peterson et al. 2011, pp. 29, 40); namely that the species has been able to disperse throughout the study region, has been at least adequately sampled, and is not inhibited from establishment due to heterogeneous biotic contexts (Anderson and Raza 2010; Barve et al. 2011; Anderson 2013; Saupe et al. 2012).

Ecological niche models: calibration and evaluation

We built models using MAXENT 3.3.1, an algorithm that has performed well in comparisons of various modeling techniques (e.g., Elith et al. 2006; Wisz et al. 2008), and produces outputs of much interest to ecologists (Elith et al. 2010, 2011). As potential environmental predictors, we used the 19 bioclimatic variables available from the WorldClim project (~1 km^2 resolution at the equator; Hijmans et al. 2005), which should be relevant for modeling aspects of the 'Grinellian niche' of these species (Luoto et al. 2007; Soberón 2010). With the aim of maximizing predictive ability under a machine learning approach, we used the complete set of variables to calibrate the models (Breiman 2001; Araújo and Guisan 2006). However, we took advantage of MAXENT's regularization to approach optimal model dimensionality and complexity (i.e., amount of variables and parameters modeling the response; Elith et al. 2011; Merow et al. 2013; see also Muscarella et al. 2014). Specifically, for each species, we produced preliminary models with various combinations of MAXENT settings and evaluated their predictive performance using

spatially independent splits of the mainland data (Peterson et al. 2011, p. 161; Radosavljevic and Anderson 2014). After determining settings resulting in the highest average predictive performance (see below), we built a model for each species using those settings and all mainland records. This model was later projected onto the peninsula. For *Heteromys anomalus*, we built a model using the settings deemed as optimal for the same dataset by Soley-Guardia et al. (2014).

Specifically, we created preliminary models for *Proechimys guairae* and *Rhipidomys venezuelae* varying two important settings from MAXENT that together affect both the dimensionality of the model (i.e., how many of the given variables are *actually* incorporated), and its complexity (i.e., how many parameters are necessary to model the response to each of the incorporated variables). This is controlled by the types of feature classes that the user allows the program to consider, and the level of regularization applied to each (Phillips et al. 2006; Merow et al. 2013). The former pertains to the types of transformations applied to the raw variables, which allow the model to explore and fit responses of various shapes to a single variable (e.g., linear, quadratic, or threshold-dependent), each response being determined by a given parameter. The latter determines how close of a match is required between the modeled responses and the empirical values (i.e., those given by the occurrence records). In this way, regularization controls both dimensionality and complexity by penalizing inclusion of variables (or response types) that do not result in substantially higher performance (i.e., model gain). Essentially, higher regularization values result in stronger penalties to complexity, reducing the potential for overfitting to any noise or bias present in the dataset (Anderson and Gonzalez 2011; Shcheglovitova and Anderson 2013).

For each species, we built the preliminary models using two different sets of feature classes under a variety of regularization values. One set consisted of those feature classes suggested by default (Phillips and Dudík 2008), while the other consisted of one in which we either added or removed feature classes (depending on whether the sample size was near that recommended by MAXENT for the use of more complex feature classes; see below). To vary the level of regularization, we modified the regularization multiplier value (hereafter regularization multiplier), which simultaneously affects the regularization coefficient (β_j) assigned to each j feature class by default (Phillips et al. 2006; Phillips and Dudík 2008; Anderson and Gonzalez 2011). Specifically, we varied the regularization multiplier every 0.5 interval between 0.5–4.0 (default settings use a regularization multiplier of 1). For *Proechimys guairae*, which had a sample size of 56 occurrence records, the default set of feature classes consisted of *linear, quadratic*, and *hinge*. We also employed a more 'complex set' adding the *threshold* and *product* feature classes (usually recommended for sample sizes > 80; see MAXENT 'Help' file). For *Rhipidomys venezuelae*, with a sample size of 22 occurrence records, the default settings included the *linear*, *quadratic*, and *hinge* feature classes. Since this was a small sample size, we also tried a simpler set, consisting of the *hinge* feature class only (which infers linear responses with potential hinges; Phillips and Dudík 2008; Merow et al. 2013). For *Heteromys anomalus*, tuning experiments were conducted in Soley-Guardia et al. (2014) using the same procedure as the present study. Those authors tested the default set, consisting of all available feature classes for continuous variables (for a sample size of 126), plus a simpler one consisting only of features classes recommended for sample sizes smaller than 80 (*linear, quadratic,* and *hinge*). Both sets of feature classes were also tested under the 0.5–4.0 range of regularization multiplier values.

For all models, we used the logistic output of MAXENT (Phillips and Dudík 2008), and kept all other settings as default (e.g., maximum number of 10,000 background points; maximum of 500 iterations). The interpretation of the logistic output of MAXENT has experienced recent critique concerning the assumption it makes about ‘prevalence’ (Royle et al. 2012; Hastie and Fithian 2013; Renner and Warton, 2013; Merow and Silander 2014). However, its use in the present study is not problematic for several reasons. First, we do not interpret the models as probability of occurrence, but rather as indices of relative suitability (i.e., relative occurrence rates; Fithian and Hastie 2013; Phillips and Elith 2013; Merow and Silander 2014). Second, the evaluation statistics employed here do not vary across MAXENT outputs, which preserve relative ranks (Phillips and Dudík 2008; Merow et al. 2013). Third, we interpret outputs independently for each lineage studied. The latter avoids the problem of determining the actual values of ‘prevalence’ for each species (necessary to compare values of absolute probability of occurrence across species; Fithian and Hastie 2013; Phillips and Elith 2013; Merow and Silander 2014). We ran models either directly from the MAXENT interface, or from batch files of command-line arguments written in JAVA (see MAXENT tutorial).

We evaluated the performance of preliminary models on spatially independent subsets of the data. This method reduces inflation of evaluation scores, typically occurring due to artifactual spatial autocorrelation between calibration and evaluation data, especially deriving from sampling biases across geography (Veloz 2009; Hijmans 2012; Wenger and Olden 2012). To do this, we used a ‘masked geographically structured *k*-fold cross validation approach’ (Radosavljevic and Anderson 2014). Specifically, we divided occurrence records into geographically structured ‘folds’ or ‘bins’ (i.e., subsets of records with a corresponding subset of the study region associated to them). We sequentially built models using all but one of these

bins, which was withheld for evaluation (i.e., neither occurrence records nor background information were drawn from that region). We achieved this by masking the geographic region corresponding to the evaluation bin during each iteration (in order to avoid violating assumptions implicit in selection of the study region; Anderson and Raza 2010; Barve et al. 2011). We defined bins longitudinally, dissecting the geographic background associated with each set of records by the middle of the longitudinally closest records of adjacent bins. For each species, the number of bins chosen corresponded to one appropriate for using the same feature classes when excluding one bin, as when using all records for the final model (suggested by MAXENT according to sample size; Phillips and Dudík 2008). While multiple options were available, we chose a number that did not result in overly narrow geographic areas (i.e., to maintain spatial independence). In this way, we used six bins for *Proechimys guairae* (each with 9–10 records), and four for *Rhipidomys venezuelae* (each with 5–6 records). For *Heteromys anomalus,* Soley-Guardia et al. (2014) used eight bins, each with 15–16 records.

As our criteria for evaluating performance of preliminary models, we used omission rates and values for the area under the curve (AUC) of the receiver operating characteristic plot (ROC) (Fielding and Bell 1997; Peterson et al. 2011, p. 162). For each iteration, omission rates were calculated over the masked bin only (withheld from calibration), whereas AUC values were calculated over the totality of the study region (as comparisons of this measure with presence-background data are only valid across the same region; see Lobo et al. 2008; Peterson et al. 2011, p. 171). We used omission rate as our principal criterion, as this tests the predictive capacity of the model regarding known data (i.e., occurrence records). Hence, it allows selection of settings that minimize overfitting to the calibration data (Shcheglovitova and Anderson 2013; Radosavljevic and Anderson 2014). Complementarily, AUC provides a *relative* measure of the

discriminatory ability of the model. However, in presence-background models, discrimination is gauged using data for which the truth is unknown (background pixels). In this way, models that assign occurrence records higher suitability values than background records are rewarded with a higher AUC value, regardless of whether the former truly hold better conditions for the species (Merow et al. 2013). For this reason, we only use this measure as a secondary criterion to assess discriminatory performance of models resulting in essentially equal omission rates. To obtain binary predictions necessary to calculate omission rates, we used the lowest presence and 10th percentile thresholds (Pearson et al. 2007; respectively the minimum training presence and 10 percentile training omission of MAXENT). We deemed as co-optimal, settings that, when averaged across all bins, resulted in an omission rate closest to that theoretically expected at both thresholds (i.e., zero for the lowest presence threshold, 10% for the 10th percentile threshold). Among co-optimal settings, we chose as optimal those with the highest AUC value, unless the difference was small (arbitrarily defined as < 0.01), in which case we chose the settings with a lower regularization multiplier value (as this usually enhances model discrimination).

Results of these preliminary models showed that settings deemed as optimal did not correspond to those suggested by default (Tables S1, S2; although the latter performed relatively well for *Proechimys guairae*). In essence, omission rates decreased for both ‘simple’ and ‘complex sets’ of feature classes as regularization increased. However, AUC values either consistently increased or decreased with increasing regularization, depending on the species. Optimal settings consisted of the ‘simple set’ of feature classes for both lineages. However, ‘complex sets’ employing high regularization also exhibited evaluation scores as good as those of simpler sets (see Soley-Guardia et al. 2014 for parallel patterns in models for *Heteromys anomalus*).

Using settings deemed as optimal in these evaluations, we built a model for each species with all records. The number of variables and parameters that were actually incorporated by MAXENT in the final model of each species are shown in Table S3. This information was obtained from the 'lambdas' file produced by each model, and it does not equate to the 'percent contribution' or 'permutation importance' provided by MAXENT (which respectively indicate the contribution of each variable during internal iterations leading to a MAXENT model, and how much a single variable affects training AUC in this model; see MAXENT tutorial). The information is also provided for the models built using the same settings but excluding the subset of records from protruding spatially marginal (PSM) localities (see below).

Table S1. Average evaluation scores of preliminary ecological niche models for *Proechimys guairae* calibrated in MAXENT with various settings. Best-performing settings are shown in bold. Averages correspond to the evaluations obtained in each of the six models made with spatially independent subsamples for each combination of settings. LQH denotes the 'simple set' of feature classes suggested by default (*linear*, *quadratic*, and *hinge*); LQHTP denotes the 'complex set' (*linear, quadratic*, *hinge*, *product*, and *threshold* feature classes).

SET OF FEATURE CLASSES	REGULARIZATION MULTIPLIER VALUE	EVALUATION OMISSION RATE (LOWEST PRESENCE THRESHOLD)	EVALUATION OMISSION RATE (10TH PERCENTILE THRESHOLD)	EVALUATION AUC (CALCULATED OVER THE ENTIRE STUDY REGION)
LQH	0.5	0.28	0.37	0.7538
LQH	1	0.13	0.28	0.7462
LQH	1.5	0.13	0.28	0.7351
**LQH**	**2**	**0.07**	**0.20**	**0.7306**
LQH	2.5	0.09	0.22	0.7277
LQH	3	0.09	0.22	0.7199
LQH	3.5	0.07	0.18	0.7096
LQH	4	0.07	0.18	0.6952
LQHTP	0.5	0.31	0.56	0.7387
LQHTP	1	0.11	0.43	0.7198
LQHTP	1.5	0.09	0.28	0.7111
LQHTP	2	0.09	0.20	0.7021
LQHTP	2.5	0.06	0.20	0.6982
LQHTP	3	0.06	0.18	0.6914
LQHTP	3.5	0.04	0.18	0.6824
LQHTP	4	0.04	0.18	0.6642

Table S2. Average evaluation scores of preliminary ecological niche models for *Rhipidomys venezuelae* calibrated in MAXENT with various settings. Best-performing settings are shown in bold. Averages correspond to the evaluations obtained in each of the four models made with spatially independent subsamples for each combination of settings. LQH denotes the 'complex set' of feature classes suggested by default (*linear*, *quadratic*, and *hinge*); H denotes the 'simple set' (*hinge* feature class only).

SET OF FEATURE CLASSES	REGULARIZATION MULTIPLIER VALUE	EVALUATION OMISSION RATE (LOWEST PRESENCE THRESHOLD)	EVALUATION OMISSION RATE (10TH PERCENTILE THRESHOLD)	EVALUATION AUC (CALCULATED OVER THE ENTIRE STUDY REGION)
LQH	0.5	0.34	0.48	0.7476
LQH	1	0.22	0.26	0.7745
LQH	1.5	0.22	0.26	0.7471
LQH	2	0.26	0.26	0.7405
LQH	2.5	0.17	0.22	0.7475
LQH	3	0.17	0.17	0.7539
LQH	3.5	0.13	0.13	0.7654
LQH	4	0.13	0.13	0.7833
H	0.5	0.34	0.52	0.7073
H	1	0.22	0.26	0.7503
H	1.5	0.13	0.17	0.7605
H	2	0.09	0.22	0.7642
H	2.5	0.09	0.13	0.7717
**H**	**3**	**0.04**	**0.13**	**0.7882**
H	3.5	0.04	0.09	0.7907
H	4	0.04	0.09	0.7962

Table S3. Details of the final ecological niche models calibrated for each species using settings deemed as optimal in the preliminary models. These correspond to the models built using all records, as well as the models built excluding records from protruding spatially marginal (PSM) localities. The parameters correspond to the features that were retained by the model (i.e., having a non-zero coefficient in the 'lambdas' file produced by MAXENT). ***n*:** sample size.

SPECIES	NUMBER OF VARIABLES INCORPORATED INTO THE MODEL (FROM A POSSIBLE TOTAL OF 19)	NUMBER OF PARAMETERS (MODELING THE RESPONSE TO EACH INCORPORATED VARIABLE)
Proechimys guairae (all records); *n* = 56<br>Features explored: linear, quadratic, and hinge<br>Regularization multiplier = 2.0	14	20
Rhipidomys venezuelae (all records); *n* = 22<br>Features explored: hinge<br>Regularization multiplier = 3.0	5	8
Rhipidomys venezuelae (without PSM subset); *n* = 20<br>Features explored: hinge<br>Regularization multiplier = 3.0	4	6
Heteromys anomalus (all records); *n* = 126<br>Features explored: linear, quadratic, and hinge<br>Regularization multiplier = 2.5	14	16
Heteromys anomalus (without PSM subset); *n* = 111<br>Features explored: linear, quadratic, and hinge<br>Regularization multiplier = 2.5	15	25

Detection and exclusion of records from protruding spatially marginal localities

In order to detect PSM localities, we followed the approach suggested by Soley-Guardia et al. (2014), who developed it using one of the species included in the present study, *Heteromys anomalus*. Specifically, we ranked occurrence records of *Proechimys guairae* and *Rhipidomys venezuelae* according to the prediction values received by the MAXENT model made with optimal settings and all mainland records (Fig. S1). Given that the five lowest-ranking records in both species spanned two major gaps in suitability values, we retrieved descriptive habitat information for these, following results of Soley-Guardia et al. (2014).

This procedure detected two records at PSM localities for *Rhipidomys venezuelae,* but none for *Proechimys guairae* (Table S4). Given that in both lineages several of these lowest-ranking records were not associated with PSM localities, we did not assess whether additional records from PSM localities existed in the dataset at higher ranks (i.e., assuming that if they did exist, they did not affect the model through issues related to their spatial marginality; see main text). This simple approach led to detection of records responsible for substantial changes in estimates of suitability across the study region, which would have otherwise lead to wrongful conclusions regarding potential for connectivity among known and hypothetical populations (Fig. S2; also Fig. 2 of main text).

However, we recognize that the approach of excluding only the records from PSM localities that received lower rankings than those not associated to PSM localities might present some limitations. Namely, because of MAXENT's machine-learning approach, the relative rank given to each record can vary according to the exact dataset used to calibrate the model (see also main text). This occurs because the actual variables and parameters incorporated into each model

can change according to the specific occurrence dataset used (e.g., including vs. excluding records from PSM localities). In this way, records from PSM localities that did not affect the first model (i.e., variables most affected by issues of spatial marginality there were not incorporated into the model), might affect the second model once it is parameterized again (i.e., variables most affected by issues of spatial marginality now become incorporated).

This issue could have been responsible for the still unrealistic prediction of the second model built for *Heteromys anomalus* under the lowest threshold, in comparison with that built for *Rhipidomys venezuelae*. For instance, for *R. venezuelae*, removal of records from PSM localities resulted in a simpler model than the one built with all records, whereas the converse was true for *H. anomalus* (Table S3). Furthermore, for the latter species, Soley-Guardia et al. (2014) reported four additional records from PSM localities receiving higher ranks than records not associated to PSM localities. In the second model built for that species, the relative ranking of records changed; however, the lowest one still corresponded to a record not associated to a PSM locality.

Projections of ecological niche models

Interpreting ENMs in novel environmental conditions (i.e., model extrapolation) is a significant challenge and an area in great need of research (Anderson 2013; Owens et al. 2013). In this study, for every model projected onto the peninsula, we took advantage of several MAXENT outputs to assess whether there were any issues associated to model extrapolation. These outputs consisted of the modeled response curves produced in the 'html' file, as well as the 'clamping',

‘multivariate similarity surface’ (MESS), and ‘most dissimilar variable’ (MoD) analyses (Elith et al. 2010).

Specifically, we first assessed whether response curves to variables with the highest contributions to the model (gauged by the ‘percent contribution’; see MAXENT tutorial) were close to achieving minimum or maximum suitability within the calibration datasets (0 or 1, respectively, for the logistic output). When modeled responses have plateaued near maximum or minimum values, different ways of extrapolating them should lead to similar conclusions, as suitability can only vary slightly. In most instances this was the case, a condition also evident in the clamping analyses (the latter consisting of zero values only). This analysis shows whether two different ways of extrapolation—suitability is assumed to remain the same for novel values (clamping) or to continue responding following the same trend (no-clamping)—lead to different estimates of suitability (see MAXENT tutorial).

However, the fact that different ways of extrapolating do not yield different results, by no means guarantees that either way is correct (regarding estimates of suitability). This is particularly true when the response curves have plateaued near maximum values, as this response has to decrease at certain point (Anderson 2013). For this reason, we also examined results from the MESS analyses. These revealed that environments within the peninsula are at most as novel as those existing within the calibration region but not incorporated during calibration (i.e., showing blue or white, but never red colors; see MAXENT tutorial). Moreover, the MoD analyses revealed that when environments were slightly novel in the peninsula (according to the MESS analyses), this novelty was never due to the variables with highest ‘percent contribution’ during calibration.

For these reasons, we infer that issues associated with model extrapolation had little effect in the results of the present study. We acknowledge that these analyses only consider novel environmental values, rather than novel environmental *combinations* (Owens et al. 2013). However, the issue of interactions among variables in general is one that clearly needs further exploration within the field of ENM, outside the scope of the currently available tools for MAXENT.

Transforming the models into categorical predictions of suitability

To test the experimental predictions, we transformed the continuous estimates of suitability given by MAXENT into categorical ones (continuous predictions shown in Fig. S2). We did so by applying two thresholds related to suitability values assigned to particular *mainland* records. Specifically, we first used the lowest presence threshold (Pearson et al. 2007; minimum training presence of MAXENT), which deems as suitable any pixel that received a value equal to or higher than the least-suitable record used to calibrate the model (i.e., even if the suitability assigned to a known record is very low, it should be sufficient for the species, given that it was found there). However, to reduce oversimplification, we also applied a stricter threshold denoting areas of higher suitability. Whereas multiple values can suit this purpose (e.g., excluding the 10th or 25th percentiles of calibration records), we chose one that at the same time would allow us to assess whether the effect of PSM localities could be reduced simply by applying a stricter threshold in the original model. Specifically, we used the value given to the lowest-ranking record that did not represent a PSM locality; hence, this threshold deems unsuitable areas that received values as low as those assigned to records from PSM localities. In this way, for *Rhipidomys venezuelae*,

where we detected two records from PSM localities, the stricter threshold corresponded to the 9th percentile. For *Heteromys anomalus,* Soley-Guardia et al. (2014) detected 15 records from PSM localities; hence, the stricter threshold corresponded to the 12th percentile. For *Proechimys guairae,* where records from PSM localities were not detected, we arbitrarily set the stricter threshold using the 9th percentile. For consistency, these percentile thresholds were also applied to the second models built without PSM localities, where they simply denote areas of higher suitability (as is also the case in the model built for *P. guairae*). The logistic values used to set these thresholds are given for all lineages in Table S5.

Figure S1. Plot used to identify the least-suitable records on the mainland, several of which represented protruding spatially marginal (PSM) localities in two of the three lineages. Records were ranked according to the logistic prediction values they obtained in the optimally parameterized MAXENT model built for each lineage. **Small black circles** represent records of *Heteromys anomalus* (from Soley-Guardia et al. 2014), **medium grey circles** those of *Proechimys guairae*, and **large grey circles** those of *Rhipidomys venezuelae*. Note that lowest suitability values were associated with occurrence at PSM localities for both *R. venezuelae* and *H. anomalus* (**hollow circles**), but not for *P. guairae*.

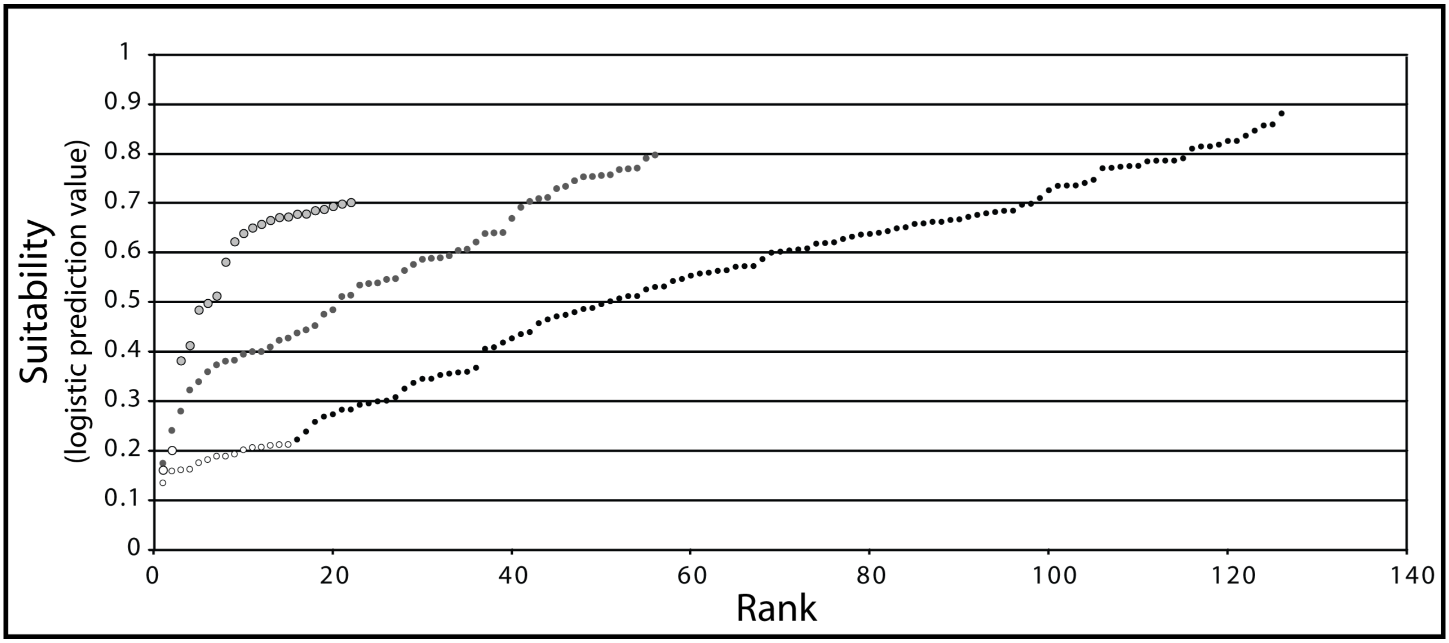

Figure S2. Estimates of suitability across the entire study region for each lineage, according to ecological niche models built including and excluding records occurring at protruding spatially marginal (PSM) localities. Projections are shown at the same scale for ease of interpretation, but note different extents for the study regions among the three lineages. For each lineage, **dark grey** indicates unsuitable areas, whereas **colors** denote suitable areas (at the lowest presence threshold). **Darker colors** indicate progressively higher degrees of suitability (in intervals of 0.1 according to the logistic prediction values given by MaxEnt). The **tan color** in the composite predictions indicates areas considered as suitable only by the model built with all records (at the lowest presence threshold). Composite predictions were constructed by overlaying the continuous estimate of suitability from the model calibrated excluding records from PSM localities on top of the binary estimate of suitability (suitable or not at the lowest presence threshold) of the model calibrated with all records (tan color). **Symbols** indicate occurrence records for each lineage; models were made with mainland records only. **Triangles** denote records occurring within PSM localities, both in the mainland and the peninsula. Note how composite predictions are much more restrictive regarding the areas they deem as truly suitable to the species (colors), but at the same time grant the potential for the species' presence at PSM localities within hotter and drier regions (tan color) *if* locally mesic conditions exist (a distinction not made by the model built with all records). These latter regions are characterized by vast expanses of xerophytic vegetation, such as thorn and desert scrub (e.g., coastal areas in and near the Península de Paraguaná, and within Península de la Guajira, west of Paraguaná), or by extensive savannas (e.g., Venezuelan *llanos* to the south; IGAC 2003; IGVSB 2004).

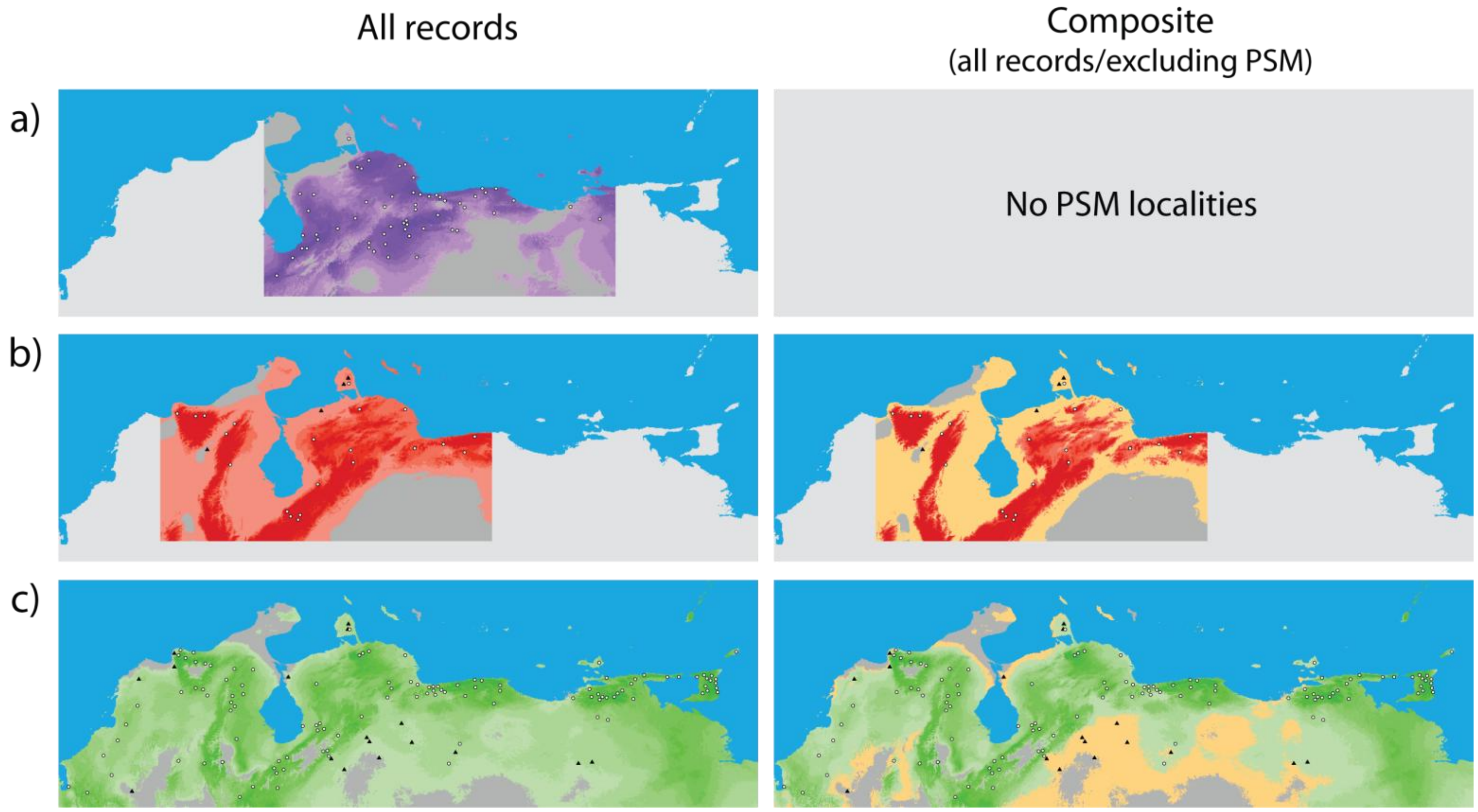
All records
Composite
(all records/excluding PSM)
a)
No PSM localities
b)
c)

Table S4. Habitat information used to determine whether inspected records corresponded to protruding spatially marginal (PSM) localities. Quotes indicate verbatim text.

RANK	LOCALITY	HABITAT DESCRIPTION	CLASSIFICATION
Proechimys guairae (mainland)			
1	Venezuela: Anzoátegui; Cueva del Agua, 435 m	Semi-deciduous dry lowland forest. Microclimate within the vicinity of the cave is slightly more humid due to small stream that runs into the cave (Aguilera, M. in litt.; translation by MSG).	Non-PSM
2	Venezuela: Miranda; La Horqueta (Tiara), 1127 m	At ca. 1100 m, the vegetation corresponds to semi-deciduous forests with patches of shade-grown coffee. (Soriano, P. in litt.; translation by MSG).	Non-PSM
3	Venezuela: Carabobo; near Montalbán, 675 m	"… [locality 36] includes both mountains and valleys surrounding Montalbán. Most of the plant growth is secondary except that in the mountains … located in a very fertile agricultural valley with oranges by far the most abundant…" Localities around Montalbán at this approximate elevation are described as having big to huge trees, sometimes interspersed with plantations (Tuttle, A.L. field notes 1967]. "[Montalbán] Lower mountain slopes with patches of mature evergreen forest, 9–18 m high; elsewhere second-growth forest and [anthropogenic] scrub dominant (Handley 1976)	Non-PSM
4	Venzuela: Miranda; Valle de Sartenejas, 1136 m	Forest intermixed with shade-grown coffee (Aguilera, M. in litt.; translation by MSG).	Non-PSM
5	Venezuela: Yaracuy; Urachiche, 869 m	Trapping took place on the mountain's slopes and above, where the vegetation corresponded to semi-deciduous or evergreen forests (Ojeda, M. in litt.; translation by MSG).	Non-PSM
Rhipidomys venezuelae (mainland)			
1	Colombia: Cesar; El Orinoco, Río César, 158 m	"Tropical forested floodland area with very large open pasture on both banks." "[Guaimaral, Rio Cesar] A caño or channel west of the main channel of the Rio Cesar. Camp about 5 kilometers west of El Orinoco. Heavily forested and more frequently flooded than the El Orinoco area" (Hershkovitz 1947).	PSM
2	Venzuela: Falcón; Capatárida, 40 m	Place is extremely dry with a vegetation dominated by different types of cacti. However along ponds and dry washes the vegetation is composed mostly by large trees and vines that are absent from the rest of the landscape. In some of these gallery forests there are also abundant cacti, but not in others. The specimen was captured in a tree within one of the local evergreen forest patches (Tuttle, A.L. field notes 1968; SVP data card].	PSM
3	Venezuela: Falcón; La Pastora, 14 km ENE of Mirimire, 190 m	"Evergreen forest located on the steep north facing slope of the mountain. At this time [November] the forest s very wet…. Grass grows in the open areas… The lower canopy is 10 m tall. The upper about 25–30 m… Many epiphytes and lianas present". The specimen was captured within the forest (Peterson, N.E. field notes 1967; SVP data card).	Non-PSM
4	Venezuela: Zulia; Misión Tukuko, 200–400 m	"A small agricultural settlement... located at about 300 m elevation... The entire region surrounding the mision was covered by evergreen rain forests as lately as 40 or 50 years ago, but most level ground has since	Non-PSM

		been cleared for cultivation or animal husbandry... Dense thickets of secondary vegetation, consisting mostly of small trees, shrubs, *Heliconia*, large aroids, and cane, occur along small streams and in swampy valley bottoms.... The canopy trees are very tall, perhaps 40 m on average and the largest are massively buttressed. Woody lianas and many subcanopy trees also remain" (Voss 1991, p. 68–70). Tall evergreen forest, only patches are anthropogenic (Voss R.S. field notes 1986). *Rhipidomys* was collected on trees in the forest, its border with coffee plantations, or on coffee plantations (Kafka, H.L. field notes 1986; Torrealba, I. field notes 1986).	
5	Colombia: La Guajira; Sierra Negra, E of Villanueva, 1500 m	"[Sierra Negra] Overlooking the town of Villa Nueva in the semiarid Cesar valley. The well drained slopes of the range from 1000–1500 m are given almost entirely to coffee. Higher up 1500–3000 m and eastward [into] Venezuela, virgin rain forests prevail (Hershkovitz 1947)". [Villanueva] "the primitive forest cover has been reduced to small isolated stands, scattered trees, shrubs and thinly wooded stream banks" (Hershkovitz 1960).	Non-PSM
Peninsular localities			
Venezuela: Falcón, Península de Paraguaná; ca. 4 km N Santa Ana, Cerro Santa Ana, 330–700 m (*Heteromys oasicus*, *Proechimys guairae*, and *Rhipidomys venezuelae*)		"the upper slopes of Cerro Santa Ana hold cloud forest and other mesic vegetation above approximately 550 m, a low proportion of its surface… Xerophytic thorn forest occurs up to 300 m. From there, deciduous montane forest extends to 550 m, where it is replaced by evergreen cloud forest. Finally, dwarf mesic vegetation occupies the remaining 150 m of the cerro. Areas above 550 m are mesic with well-developed humid soils, due to condensation of water coming of the ocean and, secondarily, by the presence of several springs above 700 m" (Anderson 2003).	Non-PSM
Venezuela: Falcón, Península de Paraguaná; San José de Cocodite, Reserva Biológica Monte Cano, 200 m (*Heteromys oasicus* and *Rhipidomys venezuelae*)		"low ridge that is predominately covered by thorn forests but that also includes relatively mesic vegetation along a seasonal watercourse… There, suitable habitat for the species exists due to mist coming off the ocean, surrounded by a matrix of xerophytic habitat on the remainder of the Península de Paraguaná... The most common vegetative formations belong to dry and very dry tropical forest, with denser formations in the gallery forests or streambeds. Due to its proximity to the sea, mist accumulates in the morning hours although it has no permanent rivers or streams... Ángela Martino-G. kindly provided unpublished information regarding the only known specimens of *Heteromys oasicus* from Monte Cano. All were captured among terrestrial bromeliads in the bed of a seasonal stream, which was dry at the time. This small stream, which is the only significant watercourse in Monte Cano reserve, flows through deciduous forest; however, trees along the stream form a semideciduous formation that maintains shade throughout the year… Traps were also placed in the reserve outside the streambed, but they yielded no *Heteromys*" (Anderson 2003).	PSM
Venezuela: Falcón, Península de Paraguaná; 49 km N, 33 km W of Coro, Moruy, 90 m (*Heteromys oasicus*)		"Our camp in Península de Paraguaná is located in Moruy, a small town located at the western base of Cerro Santa Ana. The area around camp is a dry thorn forest… Area #11, Alt. 90m [where the specimen was captured]: A desert scrub area as in #10 [see below]. The cacti are not as prominent here but the trees are more dense being about 3 m apart and averaging 10–12 m tall. In addition to the thorn trees there are some fruit trees and other [trees with] succulent thick leaves... The low shrubs also occur here but mixed in are some terrestrial bromeliads. The dry stream of area #10 occurs here but there are standing pools of water with a great deal of moss	PSM

	over the top… Area #10: Alt. 80 m: A desert scrub area with a sandy clay soil. Many thorn trees (*Mimosa*) and cactus (*Opuntia* sp., *Cereus* sp.). The ground is covered with many small to medium sized rocks. The ground cover is a type of low shrub (about 1 m tall) which is quite dense in some areas. The *Opuntia* occurs in many large clusters up to 30–500 m of pure *Opuntia*. The column cactus is scattered throughout the area. The thorn trees were scattered about 10–15 m apart and are about 8–9 m tall... The area follows a dry streambed, which has may fallen dry logs and bushes in it" (Peterson, N.E. field notes 1968)<br>.	
Venezuela: Falcón, Península de Paraguaná; 48 km N, 46 km W of Coro (Yabuquiva), 13 m<br>(*Rhipidomys venezuelae*)	This locality corresponds to a local aquifer with a higher tree density that results in locally cooler conditions. The trees are covered with vines growing along the canopy, which grant the place its local name 'La Cueva de Yabuquiva' [i.e., the cave] (Ochoa-G., J. in litt.; translated by MSG). "A thorn forest-desert scrub area, with a lot of medium sized succulent leaved trees. There are two windmills [used to extract water by the locals] and scattered pools of water occur in the area. Very little ground cover. The trees are leafless up to about 1.5 m. It looks as if the water level gets that high during part of the year. Only a few thorn trees and *Opuntia* are found here. The soil is a clay sand. No rocks could be found... The pools of water are used as watering places... Area #24 [on the other side of the road from #19]... A thorn forest-desert scrub area west of area# 19... Thorn trees, *Opuntia*, column cactus, and some low shrubs and grasses can be found but not the succulent leaved trees of area #19. The thorn trees are about 5 m apart and about 8 m high. There are no water holes in this area" (Peterson, N.E. field notes 1968).	PSM

Table S5. Thresholds used in each species to transform the continuous estimates of suitability of the MAXENT models into categorical ones. The 'lenient threshold' corresponds to the lowest presence threshold of MAXENT and denotes all areas suitable to the species; the 'stricter threshold' is a percentile threshold that denotes areas of higher suitability (see text for details). **PSM:** protruding spatially marginal localities.

SPECIES	LENIENT THRESHOLD	STRICTER THRESHOLD
Proechimys guairae		
All records	0.175	0.359
Rhipidomys venezuelae		
All records	0.161	0.382
Excluding PSM localities	0.272	0.355
Heteromys anomalus		
All records	0.135	0.222
Excluding PSM localities	0.098	0.260

Gazetteer and specimens examined from the Península de Paraguaná, outside Cerro Santa Ana

Here we report all specimens examined from Península de Paraguaná, exclusive of Cerro Santa Ana (specimens from the latter are reported in Anderson et al. 2012). Secondary information deriving from sources other than the collector is mentioned within brackets, followed by the source when applicable. Localities are arranged from north to south. For each entry, boldface type indicates the place name to which geographic coordinates correspond. We estimate that most coordinates have a maximum error of < 0.5 km; numbers of decimals vary according to the source used for georeferencing. **EBRG:** Museo de la Estación Biológica de Rancho Grande, Aragua; **MHNLS:** Museo de Historia Natural La Salle, Caracas; **USNM:** United States National Museum of Natural History, Washington, District of Columbia, USA.

FALCÓN

1. Península de Paraguaná, San José de Cocodite, **Reserva Biológica Monte Cano** [= Paraguaná, Pueblo Nuevo, Montecano; = Península de Paraguaná, Montecano], 200 m [11° 56.880’N, 69° 57.814’W; from GPS readings; based on description of sampling site; A. Martino, pers. comm.]. *Heteromys oasicus*: EBRG 20697–20698, 24378; MHNLS 11148–11151. *Rhipidomys venezuelae*: EBRG 24378.

2. 49 km N, 33 km W of Coro, **Moruy**, 90 m [11° 49.4’ N, 69° 58.3’W; DCN (1974); Peterson, N.E., field notes, 1968; not Anderson 2003]. *Heteromys oasicus*: USNM 456324.

3. 48 km N, 46 km W of Coro **(Yabuquiva)** [= Yabuquiva], 13 m [11° 48.0’ N, 70° 04.0’ W; DCN (1962); SAGCN (1990); Peterson, N.E., field notes, 1968]. *Rhipidomys venezuelae*: EBRG 15178–15179.

References

Aguilera, M., O. A. Reig, and A. Pérez-Zapata. 1995. G- and C-banding karyotypes of spiny rats (*Proechimys*) of Venezuela. Rev. Chil. Hist. Nat. 68:185–196.

Anderson, R. P. 2003. Taxonomy, distribution, and natural history of the genus *Heteromys* (Rodentia: Heteromyidae) in western Venezuela, with the description of a dwarf species from the Península de Paraguaná. Am. Mus. Novit. 3396:1–43.

Anderson, R. P. 2013. A framework for using niche models to estimate impacts of climate change on species distributions. Ann. N. Y. Acad. Sci. 1297:8–28.

Anderson, R. P., and I. Jr. Gonzalez. 2011. Species-specific tuning increases robustness to sampling bias in models of species distributions: an implementation with MaxEnt. Ecol. Model. 222:2796–2811.

Anderson, R. P., and E. E. Gutiérrez. 2009. Taxonomy, distribution, and natural history of the genus *Heteromys* (Rodentia: Heteromyidae) in central and eastern Venezuela, with the

description of a new species from the Cordillera de la Costa. *Systematic mammalogy: contributions in honor of Guy G. Musser* (eds R. S. Voss and M. D. Carleton), pp. 33–93. Bull. Am. Mus. Nat. Hist. 331.

Anderson, R. P., and A. Raza. 2010. The effect of the extent of the study region on GIS models of species geographic distributions and estimates of niche evolution: preliminary tests with montane rodents (genus *Nephelomys*) in Venezuela. J. Biogeogr. 37:1378–1393.

Anderson, R. P., E. E. Gutiérrez, J. Ochoa-G., F. J. García, and M. Aguilera. 2012. Faunal nestedness and species-area relationship for small non-volant mammals in "sky islands" of northern Venezuela. Stud. Neotrop. Fauna Environ., 47:157–170.

Araújo, M. B., and A. Guisan. 2006. Five (or so) challenges for species distribution modeling. J. Biogeogr. 33:1677–1688.

Barve, N., V. Barve, A. Jiménez-Valverde, A. Lira-Noriega, S. P. Maher, A. T. Peterson, et al. 2011. The crucial role of the accessible area in ecological niche modeling and species distribution modeling. Ecol. Model. 222:1810–1819.

Boria, R. A., L. E. Olson, S. M. Goodman, and R. P. Anderson. 2014. Spatial filtering to reduce sampling bias can improve the performance of ecological niche models. Ecol. Model. 275:73–77.

Breiman, L. 2001. Statistical modeling: the two cultures. Stat. Sci. 16:199–231.

Broennimann, O., M. C. Fitzpatrick, P. B. Pearman, B. Petitpierre, L. Pellissier, N. G. Yoccoz, et al. 2012. Measuring ecological niche overlap from occurrence and spatial environmental data. Glob. Ecol. Biogeogr. 21:481–497.

Carleton, M. D., and G. G. Musser. 2005. Order Didelphimorphia. *Mammal species of the world*, 3rd edn. (eds D. E. Wilson and D. A. Reeder), pp. 745–1600. Johns Hopkins University Press, Baltimore, MD.

DCN 1962 Hoja 6151 (Punto Fijo), escala: 1:100.000, 2nd edn. Dirección de Cartografía Nacional, Ministerio de Obras Públicas, Caracas.

DCN 1974 Hoja 6251 (Pueblo Nuevo), escala: 1:100.000, 2nd edn. Dirección de Cartografía Nacional, Ministerio de Obras Públicas, Caracas.

Elith, J., C. H. Graham, R. P. Anderson, M. Dudík, S. Ferrier, A. Guisan, et al. 2006. Novel methods improve prediction of species' distributions from occurrence data. Ecography 29: 129–151.

Elith, J., M. Kearney, and S. Phillips. 2010. The art of modelling range-shifting species. Methods Ecol. Evol. 1:330–342.

Elith, J., S. J. Phillips, T. Hastie, M. Dudík, Y. E. Chee, and C. J. Yates. 2011. A statistical

explanation of MaxEnt for ecologists. Divers. Distrib. 17:43–57.

Fielding, A. H., and J. F. Bell. 1997. A review of methods for the assessment of prediction errors in conservation presence/absence models. Environ. Conserv. 24:38–49.

Fithian, W., and T. Hastie. 2013. Finite-sample equivalence in statistical models for presence only data. Ann. Appl. Stat. 7:1917–1939.

Handley, C. O. Jr. 1976. Mammals of the Smithsonian Venezuelan Project. Brigham Young Univ. Sci. Bull. Biol. Ser. 20:1–91.

Hastie, T., and W. Fithian. 2013. Inference from presence-only data; the ongoing controversy. Ecography 36:864–867.

Hershkovitz, P. H. 1947. Mammals of northern Colombia, preliminary report no. 1: squirrels (Sciuridae). Proc. U S Nat. Mus. 97(3208):1–46.

Hershkovitz, P. H. 1960. Mammals of northern Colombia, preliminary report no. 8: arboreal rice rats, a systematic revision of the subgenus *Oecomys*, genus *Oryzomys*. Proc. U S Nat. Mus. 110(3420):513–568.

Hijmans, R. J. 2012. Cross-validation of species distribution models: removing spatial sorting bias and calibration with a null model. Ecology 93:679–688.

Hijmans, R. J., S. E. Cameron, J. L. Parra, P. G. Jones, and A. Jarvis. 2005. Very high resolution

interpolated climate surfaces for global land areas. Int. J. Climatol. 25:1965–1978.

Hortal, J., A. Jiménez-Valverde, J. F. Gómez, J. M. Lobo, and A. Baselga. 2008. Historical bias in biodiversity inventories affects the observed environmental niche of the species. Oikos 117:847–858.

IGAC 2003 Atlas de Colombia, 5ta edn. Instituto Geográfico Agustín Codazzi, Colombia.

IGVSB 2004 Mapa Ecológico, escala 1:2.000.000. Instituto Geográfico de Venezuela Simón Bolívar, Caracas.

Kozak, K. H., and J. J. Wiens. 2006. Does niche conservatism promote speciation? A case study in North American salamanders. Evolution 60:2604–2621.

Kramer-Schadt, S., J. Niedballa, J. D. Pilgrim, B. Schröder, J. Lindenborn, V. Reinfelder, et al. 2013. The importance of correcting for sampling bias in MaxEnt species distribution models. Divers. Distrib. 19:1366–1379.

Lobo, J. M., A. Jiménez-Valverde, and R. Real. 2008. AUC: a misleading measure of the performance of predictive distribution models. Glob. Ecol. Biogeogr. 17:145–151.

Luoto, M., R. Virkkala, and R. K. Heikkinen. 2007. The role of land cover in bioclimatic models depends on spatial resolution. Glob. Ecol. Biogeogr. 16:34–42.

Merow, C., and J. A. Jr Silander. 2014. A comparison of Maxlike and Maxent for modelling

species distributions. Methods Ecol. Evol. 5:215–225.

Merow, C., M. J. Smith, and J. A. Jr Silander. 2013. A practical guide to MaxEnt for modeling species' distributions: what it does, and why inputs and settings matter. Ecography 36:1058–1069.

Muscarella, R., P. J. Galante, M. Soley-Guardia, R. A. Boria, J. M. Kass, M. Uriarte, et al. 2014. ENMeval: An R package for conducting spatially independent evaluations and estimating optimal model complexity for Maxent ecological niche models. Methods Ecol. Evol. 5:1198–1205.

Owens, H. L., L. P. Campbell, L. L. Dornak, E. E. Saupe, N. Barve, J. Soberón, et al. 2013. Constraints on interpretation of ecological niche models by limited environmental ranges on calibration areas. Ecol. Model. 263:10–18.

Paynter, R. A. Jr. 1982. Ornithological gazetteer of Venezuela. Museum of Comparative Zoology, Harvard University, Cambridge, MA.

Paynter, R. A. Jr. 1997. Ornithological gazetteer of Colombia, 2nd edn. Museum of Comparative Zoology, Harvard University, Cambridge, MA.

Pearson, R. G., C. J. Raxworthy, M. Nakamura, and A. T. Peterson. 2007. Predicting species distributions from small numbers of occurrence records: a test case using cryptic geckos in Madagascar. J. Biogeogr. 34:102–117.

Peterson, A. T., J. Soberón, R. G. Pearson, R. P. Anderson, E. Martínez-Meyer, M. Nakamura, et al. 2011. *Ecological niches and geographic distributions.* Monographs in Population Biology, 49. Princeton University Press, Princeton, NJ.

Phillips, S. J., and M. Dudík. 2008. Modeling of species distributions with MaxEnt: new extensions and a comprehensive evaluation. Ecography 31:161–175.

Phillips, S. J., and J. Elith. 2013. On estimating probability of presence from use-availability or presence background data. Ecology 94:1409–1419.

Phillips, S. J., R. P. Anderson, and R. E. Shapire. 2006. Maximum entropy modeling of species geographic distributions. Ecol. Model. 190:231–259.

Phillips, S. J., M. Dudík, J. Elith, C. H. Graham, A. Lehmann, J. Leathwick, et al. 2009. Sample selection bias and presence-only distribution models: implications for background and pseudo-absence data. Ecol. Appl. 19:181–197.

Radosavljevic, A., and R. P. Anderson. 2014. Making better MAXENT models of species distributions: complexity, overfitting, and evaluation. J. Biogeogr. 41:629–643.

Renner, I. W., and D. I. Warton. 2013. Equivalence of MAXENT and Poisson Point Process models for species distribution modeling in ecology. Biometrics 69:274–281.

Royle, J. A., R. B. Chandler, C. Yackulic, and J. D. Nichols. 2012. Likelihood analysis of

species occurrence probability from presence-only data for modelling species distributions. Methods Ecol. Evol. 3:545–554.

SAGCN 1990 Hoja 6151-II-NE (Yabuquiva), escala: 1:25.000, 2nd edn. Servicio Autónomo de Geografía y Cartografía Nacional, Ministerio del Ambiente y de los Recursos Naturales Renovables, Caracas.

Saupe, E. E., V. Barve, C. E. Myers, J. Soberón, N. Barve, C. M. Hensz, et al. 2012. Variation in niche and distribution model performance: the need for a priori assessment of key causal factors. Ecol. Model. 237:11–22.

Shcheglovitova, M., and R. P. Anderson. 2013. Estimating optimal complexity for ecological niche models: a jackknife approach for species with small sample sizes. Ecol. Model. 269:9–17.

Soberón, J. M. 2010. Niche and area of distribution modeling: a population ecology perspective. Ecography 33:159–167.

Soley-Guardia, M., A. Radosavljevic, J. L. Rivera, and R. P. Anderson. 2014. The effect of spatially marginal localities in modelling species niches and distributions. J. Biogeogr. 41:1390–1401.

Syfert, M. M., M. J. Smith, and D. A. Coomes. 2013. The effects of sampling bias and model

complexity on the predictive performance of MaxEnt species distribution models. PLoS ONE 8(2):1–10.

Tribe, C. J. 1996. *The neotropical rodent genus* ‘Rhipidomys’ *(Cricetidae: Sigmodontinae): a taxonomic revision*. PhD thesis, University College London, London.

Veloz, S. D. 2009. Spatially autocorrelated sampling falsely inflates measures of accuracy for presence-only niche models. J. Biogeogr. 36:2290–2299.

Voss, R. S. 1991. An introduction to the Neotropical muroid rodent genus *Zygodontomys*. Bull. Am. Mus. Nat. Hist. 210:1–113.

Voss, R. S., D. P. Lunde, and N. B. Simmons. 2001. The mammals of Paracou, French Guiana: a Neotropical rainforest fauna. Part 2. Nonvolant species. Bull. Am. Mus. Nat. Hist. 263:1–236.

Warren, D. L., R. E. Glor, and M. Turelli. 2008. Environmental niche equivalency versus conservatism: quantitative approaches to niche evolution. Evolution 62:2868–2883.

Warren, D. L., R. E. Glor, and M. Turelli. 2010. ENMTools: a toolbox for comparative studies of environmental niche models. Ecography 33:607–611.

Wenger, S. J., and J. D. Olden. 2012. Assessing transferability of ecological models: an underappreciated aspect of statistical validation. Methods Ecol. Evol. 3:260–267.

Wisz, M. S., R. J. Hijmans, J. Li, A. T. Peterson, C. H. Graham, A. Guisan, et al. 2008. Effects of sample size on the performance of species distribution models. Divers. Distrib. 14:763–773.
